# Innovative Insights into Traumatic Brain Injuries: Biomarkers and New Pharmacological Targets

**DOI:** 10.3390/ijms25042372

**Published:** 2024-02-17

**Authors:** Serena Silvestro, Ivana Raffaele, Angelo Quartarone, Emanuela Mazzon

**Affiliations:** IRCCS Centro Neurolesi Bonino Pulejo, Via Provinciale Palermo, SS 113, Contrada Casazza, 98124 Messina, Italy; serena.silvestro@irccsme.it (S.S.); ivana.raffaele@irccsme.it (I.R.); angelo.quartarone@irccsme.it (A.Q.)

**Keywords:** traumatic brain injury, molecular mechanisms, physiological responses, neuroprotection, rehabilitation, regenerative medicine

## Abstract

A traumatic brain injury (TBI) is a major health issue affecting many people across the world, causing significant morbidity and mortality. TBIs often have long-lasting effects, disrupting daily life and functionality. They cause two types of damage to the brain: primary and secondary. Secondary damage is particularly critical as it involves complex processes unfolding after the initial injury. These processes can lead to cell damage and death in the brain. Understanding how these processes damage the brain is crucial for finding new treatments. This review examines a wide range of literature from 2021 to 2023, focusing on biomarkers and molecular mechanisms in TBIs to pinpoint therapeutic advancements. Baseline levels of biomarkers, including neurofilament light chain (NF-L), ubiquitin carboxy-terminal hydrolase-L1 (UCH-L1), Tau, and glial fibrillary acidic protein (GFAP) in TBI, have demonstrated prognostic value for cognitive outcomes, laying the groundwork for personalized treatment strategies. In terms of pharmacological progress, the most promising approaches currently target neuroinflammation, oxidative stress, and apoptotic mechanisms. Agents that can modulate these pathways offer the potential to reduce a TBI’s impact and aid in neurological rehabilitation. Future research is poised to refine these therapeutic approaches, potentially revolutionizing TBI treatment.

## 1. Introduction

Traumatic brain injuries (TBIs) are a condition marked by structural or functional damage to the brain resulting from a traumatic event, such as an accident, fall, or violent incident, initiating a cascade of events in the brain tissue [1]. By the year 2020, TBIs were anticipated to rank as the third leading cause of death worldwide, contributing to a concerning annual toll of 1.7 million cases in the United States alone [2]. TBIs can be categorized into closed, which is a non-penetrating injury, and open-head injuries, which involve an object piercing the skull and penetrating the dura mater. The former, a non-penetrating injury, is more commonly observed than the latter [3]. 

This event triggers a molecular cascade in brain tissue. The primary phase of a TBI is marked by mechanical injury, including the stretching, compression, and tearing of the brain tissues and blood vessels. This leads to an initial cell loss, initiating a series of biochemical changes in the secondary phase of the injury [4]. From a pathological standpoint, this clinical condition involves a complex interplay of structural, neurochemical, and inflammatory changes in response to head trauma, affecting brain function and the patient’s quality of life.

Following the injury, a complex interplay of neurotransmitters, biochemical mediators, cytokines, and genetic changes contributes to the molecular mechanisms underlying tissue damage. Excitotoxicity, involving the overactivation of neurotransmitters, is a critical pathophysiological factor, along with disturbances in calcium homeostasis, nitric oxide production, and the generation of reactive oxygen species (ROS), which contribute to cell death through apoptosis [5,6]. These molecular events trigger programmed cell death pathways, exacerbating tissue damage. Injured brain tissue initiates an immune response, leading to inflammation that can both harm and potentially facilitate repair processes.

Furthermore, several investigations report a significant increase in the presence of “reactive astrocytes”, which serve as key regulators in both tissue damage and potential repair, representing a distinctive hallmark of TBIs [7,8]. Understanding the intricate molecular interactions following a TBI, involving neurons, glial cells, and vascular networks, is crucial for managing the injury and its consequences.

This type of injury can manifest in various forms and severities, with consequences that can significantly differ from case to case. The severity of TBIs is classified as mild, moderate, or severe based on clinical assessment, including the Glasgow Coma Scale (GCS). The GCS is a scale for measuring neurocognitive function, ranging from three, indicating complete unresponsiveness, to fifteen, which denotes full responsiveness. When combined with computed tomography (CT) imaging, the GCS aids in determining TBI severity and prognosis. Statistically, approximately 10% of TBI patients admitted to hospitals suffer from moderate (GCS of 9–13)-to-severe TBIs (GCS of 3–8). However, the majority, approximately 90%, experience mild TBIs (mTBIs), characterized by GCS scores of 13 to 15 [9]. The prognosis for TBIs is challenging due to their wide range of symptoms, which can vary from temporary confusion in mild cases to long-term physical, psychological, and cognitive impairments, possibly leading to coma or even death in more severe instances [10,11]. TBI outcomes are influenced by factors such as patient characteristics, injury details, and the quality of care. Research has shown that survivors of moderate and severe TBIs may experience disability one year after the injury. Moreover, epidemiological studies suggest that these TBIs could be potential risk factors for developing neurodegenerative disorders like Parkinson’s or Alzheimer’s disease, or other neurological disorders [12]. While prognostic models have been developed and validated for moderate and severe TBIs, such models are less established for mild TBIs, and there is no universal model that covers the entire TBI severity spectrum. Given the heterogeneity of TBIs and their variable clinical course, improving outcome prediction may involve incorporating new information over time or including factors that predict treatment [13].

Most patients with mTBI recover quickly, but about 15% develop persistent post-concussive syndrome, which is characterized by symptoms such as headaches, dizziness, and cognitive difficulties [14,15]. mTBIs, also known as brain concussion, once considered minor, have been recognized for its long-term neuropsychological impacts, particularly in contact-sport athletes and military personnel [16]. Studies have shown that repetitive concussions can increase the risk of chronic traumatic encephalopathy (CTE). Indeed, about 97% of cases have been recently reported in individuals exposed to repetitive head impacts, though incidence data remain unclear due to limitations in current research methodologies and the overlap of its neuropathological features with other neurodegenerative diseases [17,18,19]. This highlights the complexity and variability of the remote consequences of TBIs, such as CTE, that pose significant challenges due to their varied presentations and the long-term impact on individuals’ quality of life. Current research efforts are directed towards better understanding the epidemiology of these post-traumatic conditions, which is crucial for developing targeted interventions and support mechanisms for affected patients.

TBIs have long presented a complex challenge to researchers due to their intricate pathophysiology, varied clinical presentations, and the delicate equilibrium needed in treating brain inflammation and subsequent neuronal injury [20]. This review assessed the significance of biomarkers related to TBIs, the crucial molecular mechanisms involved, and the most recent advancements in therapeutic strategies. The methodological approach of this review is aimed at providing a comprehensive and in-depth understanding of TBIs, encompassing both traditional and innovative concepts in this area. Through a comprehensive analysis and in-depth examination of this field, this review could be useful for future studies that will explore innovative therapeutic approaches for TBIs.

## 2. Methodology

An extensive review of the literature from 2021 to the present was conducted in December 2023 using multiple databases (PubMed, Google Scholar, and ClinicalTrials.gov).

The sections titled “3. Traumatic Brain Injury Biomarkers” and “4. Advances in TBI: Molecular Mechanisms and Therapeutic Insights” were specifically compiled based on a bibliographical search using the following keywords: “Mild Traumatic Brain Injury AND Proteomic Biomarkers” OR “Neurofilament Light Chain AND Traumatic Brain Injury” OR “Ubiquitin C-terminal Hydrolase-L1 AND Traumatic Brain Injury” OR “FDA Approved and Traumatic Brain Injury Biomarkers” OR “Traumatic Brain Injury AND S100B” OR “Traumatic Brain Injury Treatment AND Neuroprotection AND Neuroinflammation AND Apoptosis” OR “Traumatic Brain Injury Treatment AND Neuroprotection AND Neuroinflammation AND Astrocytes Activation” OR “Traumatic Brain Injury Treatment AND Neuroprotection AND Neuroinflammation AND Microglia Activation” OR “Traumatic Brain Injury Treatment AND Neuroprotection AND Neuroinflammation AND NLRP3” OR “traumatic brain injury treatment and neuroprotection and oxidative stress” OR “traumatic brain injury treatment and neuroprotection and ferroptosis”.

This search was specifically focused on peer-reviewed articles published in the English language. Each article underwent an evaluation to determine its relevance in the context of TBI biomarkers, molecular mechanisms, and the latest advancements in therapeutic interventions. The objective of this review methodology is to ensure a comprehensive and multifaceted comprehension of TBIs, shedding light on both established and emerging concepts in this domain. By amalgamating diverse viewpoints from various studies, this review aims to furnish valuable insights into the present status of TBI research and its prospective trajectories. The primary goal of this review was to establish a robust groundwork for uncovering the molecular and physiological mechanisms of TBIs and gaining insights into the most recent developments in the exploration of innovative therapeutic approaches.

## 3. TBI Biomarkers

Biomarkers detected in body fluids are being considered as potential evaluation tools for patients with TBIs. They can act as indicators of cerebral damage and provide valuable information about the dynamic cellular, biochemical, and molecular environments [21]. In recent years, particularly over the last three years, research in this area has advanced significantly. Emerging evidence underscores the significance of specific blood biomarkers such as neurofilament light chain (NF-L), ubiquitin carboxy-terminal hydrolase-L1 (UCH-L1), Tau, S100B, and glial fibrillary acidic protein (GFAP) [22]. Recently, using the Breakthrough Devices Program, UCH-L1 and GFAP were approved by the Food and Drug Administration (FDA) as the first blood-based biomarker called the Brain Trauma Indicator™ (BTI™) [23].

The clinical validation of these biomarkers would be a useful prognostic tool to monitor the response to treatment [24]. This would be a valid approach aimed at improving therapy for patients with TBIs.

### 3.1. Preclinical Study

In a mouse model of mTBI caused by low-intensity blasts, significant changes were observed in NF-L and GFAP levels in brain tissues and plasma. These changes, potentially linked to increased blood-brain barrier (BBB) permeability, were also associated with structural damage to the myelin, mitochondria, and synapses in the neurovascular unit. These alterations can affect cerebral blood flow and cellular interactions, leading to increased vulnerability to brain damage and dysfunction. However, this study did not conclusively determine the primary utility of these biomarkers in diagnosis or prognosis but indicated their potential relevance in both areas [25]. Furthermore, in a repeated mTBI model, a surge in serum NF-L levels highlighted its effectiveness in detecting neuronal damage across various TBI scenarios. Unlike the previous study, which explored the impact of a single low-intensity event, here, the change in this biomarker following repeated mTBI over time was examined, only demonstrating an increase in the acute phase. Furthermore, mass spectrometry detected significant changes in 26 proteins at 7 days and in 72 proteins at 3.5 months after a mTBI. These protein variations may be implicated in neuroinflammation, energy metabolism, and neurogenic capacity, and consequently, their diagnostic and prognostic implications [26].

Another in vivo study investigated the co-occurrence of TBIs and severe blood loss leading to hemorrhagic shock. The circulating GFAP was associated with histopathological markers of diffuse axonal injury and BBB breaches, suggesting that GFAP, along with NFL and UCH-L1, is sensitive to various trauma types, not just specific pathologies [27]. This comorbidity study involving TBIs and hemorrhagic shock is particularly noteworthy for its exploration of complex clinical scenarios, but again, the translational aspect of human treatment needs more emphasis. 

These preclinical studies highlight diagnostic and prognostic potential. The findings about biomarker variations in different TBI scenarios, especially in repeated injuries, are crucial for developing diagnostic and prognostic tools. However, they also note limitations; for instance, the extrapolation of results from animal models to human conditions is always challenging and may not fully represent the human physiological responses.

### 3.2. Clinical Study

The significance of biomarkers in the clinical management of TBIs is considerable, offering enhancements in diagnosis, prognosis, and monitoring of treatment. Structural and molecular biomarkers, including indicators of neuronal, glial, and axonal damage, are instrumental in evaluating the extent of an injury and tracking processes of secondary repair. Advanced proteomic and lipidomic techniques provide fresh insights into the mechanisms of injury and repair [28]. The assessment of blood biomarkers has demonstrated potential for delivering objective measures for TBI evaluation, enhancing diagnostic and prognostic capabilities across the spectrum of TBI severity, from concussions to severe TBIs [29]. Ongoing research is delving into novel biomarkers and integrated approaches, underscoring the necessity of a multimodal assessment incorporating various biomarkers for a more precise diagnosis and monitoring of TBIs [21]. These studies underscore the pivotal role of biomarkers in refining the clinical care of TBI patients, from early and accurate diagnoses to damage assessment and recovery, and in informing therapeutic strategies.

#### 3.2.1. Biomarkers of Neuronal, Glial, and Axonal Damage in Traumatic Brain Injurys

GFAP and UCH-L1 are the first TBI biomarkers approved by the FDA for acute TBIs, as supported by several studies [30,31,32,33]. These studies not only confirm their diagnostic value but also highlight their prognostic potential in predicting mortality and identifying the patients at risk of adverse outcomes [30]. They also demonstrate the accuracy of a rapid blood-based test combining GFAP and UCH-L1 in predicting acute intracranial injuries following mTBIs. This test has been shown to have a high sensitivity (95.8%) for detecting such lesions [31]. A strong correlation was also obtained from a central laboratory platform. This test could optimize clinical decisions about CT use and reduce invasive diagnostic procedures in emergency settings [32]. These studies show that GFAP and UCH-L1 are not only useful for the initial diagnosis of TBI but can also provide valuable prognostic information and predict death and adverse outcomes, but not for the prediction of incomplete recovery at 6 months. Furthermore, their usefulness in the hyperacute phase (within the first 2 h) of a TBI was also demonstrated, and the sport-related concussion calcium-binding protein B (S100B) biomarker was also analyzed, providing a direct comparison with GFAP and UCH-L1 [33]. Sara M. Lippa et al. studied the link between blood biomarkers and cognitive decline post-TBI, categorizing a cohort by TBI severity. They discovered that initial GFAP and UCH-L1 levels strongly predicted memory decline in severe TBI, while Tau and NF-L were key indicators in mTBI. This biomarker specificity marks progress towards tailored treatments. Predicting cognitive decline through initial biomarker levels aids in selecting fitting therapies. However, larger, more varied population studies are needed to validate these results [34]. In CENTER-TBI’s extensive study with 2869 patients, six serum biomarkers (GFAP, NF-L, NSE, S100B, total Tau, and UCHL1) were tested within 24 h of an injury. Their findings showed a link between biomarker levels and brain lesion severity and size, affirming their importance in gauging injury severity, not type [35].The research on biomarkers such as GFAP, UCH-L1, and others offers a fundamental insight into their role in TBIs in various contexts. Recent studies, particularly on S100B, broaden this understanding to pediatric TBI. Notably, higher S100B protein levels were observed in patients with post-concussion syndrome, indicating a correlation between this protein and TBI severity [36]. These findings align with earlier research in adults involving biomarkers such as GFAP and UCH-L1, emphasizing the wide-ranging relevance of biomarkers across various age groups and injury severities. In a separate study, S100B was effective in predicting 6-month mortality and functional outcomes post-TBI in children. Conversely, plasma osteopontin served as an indicator of disease severity, illustrating the distinct utility of each biomarker rather than their combined application [37]. In another study, however, the focus was shifted to IL-8, highlighting this interleukin as a strong predictor of persistent fatigue 12 months after a TBI in children, adding a new perspective on using biomarkers in pediatric brain injuries [38]. Overall, these data highlight the importance of biomarkers in predicting various outcomes following pediatric brain injuries. Together, these studies reinforce the potential of biomarkers in personalized medicine for TBIs, emphasizing their importance in both diagnostic and prognostic settings across diverse patient populations.

Additionally, this review extends its scope by examining these biomarkers in more specialized contexts, including sport-related concussions and injuries sustained during military training. For example, the increase in GFAP and NF-L levels among collegiate football players, regardless of their diagnosed concussions, echoes the previous findings on the sensitivity of these biomarkers to brain injuries. This supports the idea that biomarkers can detect subclinical brain injuries in athletes, an insight that was not fully captured in earlier studies focused on more general TBI contexts. Moreover, this study revealed that players in roles requiring greater speed and high-intensity impacts, such as quarterbacks and receivers, exhibited higher Tau and NF-L levels compared to those in slower positions like linemen [39]. Similarly, the study on professional rugby players identified S100B, neuron-specific enolase (NSE), spectrin breakdown products (SBDPs), UCHL-1, GFAP, NF-L, and Tau as biomarkers. This study emphasized the nuanced roles of biomarkers like S100B and NF-L in differentiating between resolving and non-resolving trauma cases. Specifically, only S100B remained stable during the season, indicating its reliability as a biomarker of inconclusive sport-related concussion [40]. Lastly, the study involving United States military cadets further corroborates the responsiveness of GFAP and UCH-L1 to brain injuries, reinforcing their utility in diverse settings, including military training [41]. In line with these studies, Tau, NF-L, GFAP, and UCHL-1 were found elevated in 84 US military personnel and veterans prospectively enrolled in the 15-year longitudinal study. These biomarkers have been associated with the deterioration of neurobehavioral symptoms within 12 months of injury, identifying them as promising prognostic tools useful for identifying patients who have a high risk of showing positive results after a TBI [42]. The analysis of blood biomarkers in individuals exposed to repeated head impacts demonstrated that NF-L levels were higher in active boxers compared to Mixed Martial Arts fighters. Notably, GFAP levels were higher in retired boxers, with a correlation with decreased volumes of multiple brain structures and cognitive decline over time. Thus, plasma GFAP levels could be useful in identifying individuals who are at increased risk of progressive brain atrophy and cognitive impairment due to repetitive head trauma, particularly in individuals exposed to boxing-related activities [27].

#### 3.2.2. Biomarkers of Synaptic Damage and Dysfunction

Introducing beta-synuclein as a novel biomarker for synaptic damage represents a significant advancement. This study, which also included NFL and GFAP, highlights the distinct temporal patterns of biomarker levels following poly-injury trauma. The early elevation of beta-synuclein and GFAP, contrasted with the gradual increase of NFL, offers a more nuanced understanding of the biomarkers’ response to brain injuries, enhancing the ability to differentiate between patients with and without a TBI [43]. The collegiate athlete study brings another layer of complexity by analyzing a broad-spectrum of 1305 proteins. Among these, erythrocyte membrane protein band 4.1 (EPB41) and alpha-synuclein (SNCA) emerged as key biomarkers, effectively distinguishing athletes who have suffered trauma from those who have not. EPB41 and SNCA showed high sensitivity and specificity in detecting brain damage within hours of an injury [44]. In summary, these studies collectively enhance our understanding of biomarkers in brain injuries, highlighting their sensitivity to various forms of trauma, their utility in detecting subclinical injuries, and their potential in differentiating between types of brain damage in specific contexts like sports and military training.

#### 3.2.3. Extracellular Vesicles as Biomarkers with Neurofilament Light Chain (NF-L), Tau, and Glial Fibrillary Acidic Protein (GFAP) in Focus

Building on previous research on TBI biomarkers, recent studies have delved into the analysis of extracellular vesicles (EVs) to uncover low-level molecules in the blood that indicate TBI and PTSD processes. In a pivotal study, combat-exposed service members, with and without TBI history, were examined to assess 798 microRNAs in circulating EVs. Additionally, eight proteins in EVs and plasma, including NFL, Tau, amyloid beta (Aβ)42, Aβ40, interleukin-6 (IL-6), IL-10, tumor necrosis factor-alpha (TNF-α), and vascular endothelial growth factor (VEGF), were measured. This study’s results showed a link between persistent post-traumatic symptoms and levels of microRNAs in EVs, particularly hsa-miR-139-5p, which is known to be related to neurodegenerative processes. NFL was also found to correlate with the severity of post-concussion symptoms [45]. The ability of NFL to serve as a biomarker for determining the severity of head trauma was further confirmed in a study involving military personnel and veterans with various degrees of head trauma, ranging from uncomplicated to severe. This study indicated that, within one year of injury, EV NFL levels were significantly higher in individuals with more severe injuries compared to those with mTBI and healthy subjects. Although median GFAP levels were higher in individuals with severe TBIs, the difference was not significant [46]. These alterations suggest that NFL and GFAP might reflect different aspects of brain injury, with NFL possibly indicating axonal degeneration and GFAP pointing to astrocytic pathological processes. EVs therefore represent a non-invasive method to extrapolate and better understand the state of the brain, overcoming some of the limitations of traditional detection methods. The use of advanced technologies like single-molecule array (SIMOA) [47] and Track Etched Magnetic Nanopore (TENPO) technology for isolating brain-specific EV marks (GluR2+, glutamate ionotropic receptor AMPA type subunit 2) is a significant innovation in biomarker analysis. This allows brain-specific biomarkers to be differentiated from non-specific ones, creating a diagnostic panel for mTBI through ultrasensitive digital enzyme-linked immunosorbent assay (ELISA) techniques. This is important news, as biomarkers in mTBI are often difficult to identify [48].

#### 3.2.4. Novel Selective Blood Axonal Injury Biomarkers

Among these biomarkers for severe TBIs, total serum Tau protein is the best characterized. However, the measurement of serum Tau does not allow for distinguishing the peripheral one from the specific one of the central nervous system. Therefore, the research group led by Fernando Gonzalez-Ortiz et al. has identified a new selective blood biomarker for the central nervous system, Brain-derived-Tau. The serum level of Brain-derived Tau could be a valid biomarker to discriminate the severity of TBIs and monitor the clinical outcomes both on the day of damage and after 7 days [49].

**Figure 1:**
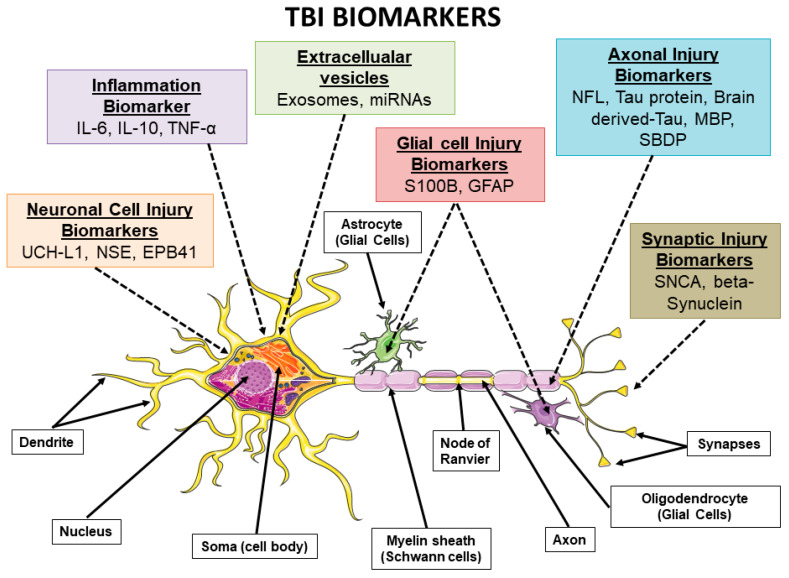
In this image, the main types of TBI biomarkers are represented, highlighting the molecular processes in which they are involved, such as neuronal damage, glial damage, axonal damage, and inflammation. Specifically, NSE, SBP, UCH-L1, and EPB41 are biomarkers linked to neuronal cell body lesions. S100-B and GFAP are injury biomarkers of astroglial cells. Post-TBI astrogliosis and neuroinflammation can cause an increase in the production of interleukins and cytokines (IL-6, IL-10, and TNF-α), which can therefore be considered TBI biomarkers. NFL, Tau protein, Brain-derived Tau, MBP, and SBDP are axonal injury biomarkers. Beta-synuclein and SNCA are blood biomarkers of synaptic damage. The image was created using the image bank of Servier Medical Art (available online: http://smart.servier.com/; accessed on 30 December 2023) licensed under a Creative Commons Attribution 3.0 Unported License (available online: https://creativecommons.org/licenses/by/3.0/; accessed on 30 December 2023). Aβ: amyloid beta; EPAB41: erythrocyte membrane protein band 4.1; EVs: extracellular vesicles; GFAP: glial fibrillary acidic protein; IL-10: interleukin-10; IL-6: interleukin-6; NF-L: neurofilament light chain polypeptide; NSE: neuron-specific enolase; S100B: sport-related concussion calcium-binding protein B; SBDP: spectrin breakdown product; SNCA: alpha-synuclein; TBI: traumatic brain injury; TNF-α: tumor necrosis factor-alpha; UCH-L1: ubiquitin C-terminal hydrolase-L1; and VEGF: vascular endothelial growth factor.

The findings of these data, summarized in Table 1, reveal significant correlations between these biomarkers and various TBI outcomes, including neurodegenerative and molecular processes (Figure 1), highlighting their potential in diagnosing, prognosing, and understanding TBIs. In conclusion, the finding that baseline levels of biomarkers such as GFAP, UCH-L1, Tau, and NF-L can predict cognitive decline in patients with TBIs of varying severity. Noteworthy, GFAP and UCH-L1 have been approved by the FDA for their use in diagnosing acute TBIs. In the acute phase of TBIs, the timeliness and accuracy of diagnosis are essential for making immediate and appropriate medical decisions. Biomarkers represent an intriguing option in this context as they can be rapidly measured from blood, providing almost instantaneous results [50]. This can be particularly valuable in emergency situations where efficiency is crucial. These biomarkers have demonstrated a high sensitivity of 95.8% in detecting acute intracranial lesions following mTBIs [51]. This implies that a rapid test based on these biomarkers could reduce the necessity for CT scans, which are more invasive and involve exposure to radiation. A combined test involving both UCH-L1 and GFAP has exhibited a high sensitivity and negative predictive value for identifying traumatic intracranial lesions on CT scans, supporting its potential clinical utility in ruling out the need for CT scans in patients presenting to emergency rooms with a suspected TBI [52]. Beyond their diagnostic role, GFAP and UCH-L1 may also offer valuable prognostic information, such as predicting mortality and adverse outcomes following TBIs. Their usefulness in the hyperacute phase (within the first 2 h) of a TBI suggests that they could be essential tools for early detection and timely intervention. The use of biomarkers during the acute phase of a TBI represents a significant advancement, providing a quicker, less invasive, and potentially more accurate diagnostic and prognostic approach compared to traditional methods. This paradigm shift has the potential to greatly enhance the care of TBI patients. However, it is important to note that biomarkers may not completely replace CT scans in all cases. CT scans provide more precise anatomical details and may be necessary to fully assess the extent of brain injuries, especially in severe TBIs. Therefore, the use of biomarkers should be seen as a complement to CT scans rather than a total replacement. Additionally, it is important to continue research to further validate the effectiveness of biomarkers in diagnosing acute TBIs and identify situations where they may be more beneficial. Furthermore, recent studies have extended this understanding to pediatric cases of TBIs, demonstrating the utility of biomarkers such as S100B and osteopontin in specific contexts. Furthermore, the use of biomarkers in more specific contexts, such as sport-related concussions and injuries during military training, highlights their sensitivity to various types of trauma. The introduction of new biomarkers such as beta-synuclein and the analysis of EVs incorporating NF-L, GFAP, and others offer new perspectives in understanding the nature and dynamics of TBIs. These studies highlight the potential of brain-specific biomarkers in differentiating between different types of brain damage and in the prospect of personalized medicine for TBIs. However, it still remains complex to identify biomarkers that can reliably distinguish between various degrees of TBI; therefore, it is necessary to continue research to fully understand their role and potential in neurology. Future research should therefore focus on exploring these biomarkers in larger and more diverse samples and further explore their involvement in post-injury temporal dynamics.

## 4. Advances in Traumatic Brain Injury (TBI): Molecular Mechanisms and Therapeutic Insights

Recent advancements in TBI research focus on understanding injury mechanisms and developing therapeutic interventions. Neuroprotection is crucial, aiming to mitigate brain damage and support regeneration by modulating biological pathways, thus minimizing neuronal damage and enhancing recovery [53]. Discovering compounds that not only reduce brain damage, neuronal loss, and microglial activation but also enhance long-term neurological functions represents a significant advancement in TBI treatment strategies. This evolving understanding has opened new avenues for effective therapies, underlining the critical role of neuroprotection in mitigating brain damage and promoting tissue regeneration in TBI research.

### 4.1. Targeting Multiple Pathways for Neuroprotection in Preclinical Traumatic Brain Injury (TBI) Studies

TBIs originate from mechanical impacts that disrupt brain structures, leading to primary injuries such as contusions, lacerations, and hemorrhages. These direct injuries adversely affect the cerebral parenchyma, compromise the BBB, and impact neurons, glial cells, and the brain vasculature [1]. After these initial events, a cascade of biochemical, cellular, and physiological changes unfolds, potentially exacerbating the primary trauma. Prompt interventions are crucial in mitigating secondary complications, like hypoxia and cerebral edema, to reduce neuronal damage and enhance recovery outcomes [54]. 

Secondary injuries in TBIs are marked by molecular disruptions, including oxidative stress, excitotoxicity, and calcium homeostasis imbalances. These alterations can further aggravate brain damage, accelerating the injury’s progression and potentially increasing the risk of cognitive decline and neurodegenerative diseases. The measurement of biomarkers can help in evaluating the effectiveness of therapeutic compounds aimed at alleviating brain damage [55]. However, biomarkers need to be correlated with clinical assessments to confirm improvements in neurological functions. Understanding the mechanisms of damage in TBIs and developing neuroprotective and restorative strategies are crucial for improving the outcomes for TBI survivors. Studies have investigated the relationship between structural brain damage and functional impairment in both experimental and clinical settings. The brain’s natural responses to trauma, involving inflammatory and neuro-restorative processes, might be insufficient to halt damage progression post-TBI [56]. 

Research indicates that female mice have better memory retention after a TBI, potentially due to hormonal fluctuations during their estrous cycle [57]. In this context, tibolone, a synthetic hormone, has shown its potential for neuroprotection against post-TBI inflammation and oxidative stress, likely through estrogen receptor activation [58].

#### 4.1.1. Anti-Inflammatory Agents and Immunomodulators in Traumatic Brain Injury (TBI) 

Following a TBI, the activation of microglia—the brain’s resident immune cells—and the infiltration of macrophages from the periphery are essential in supporting neuronal survival and aiding recovery in and around the injury site. This immune response engages in several protective mechanisms, including the phagocytosis of damaged cells, the release of cytokines and neurotrophic factors, and the initiation of tissue repair [59,60]. The precise mechanisms underlying neural recovery post-TBI remain not fully understood. 

Recent research underscores the therapeutic potential of modulating the infiltration of peripheral cells and the activation of glial cells, particularly microglia and astrocytes, in TBI. Treatments that reduce the levels of pro-inflammatory molecular biomarkers, GFAP and the ionized calcium-binding adaptor molecule 1 (Iba1), have been associated with a lower inflammatory response, leading to the mitigation of injury-related deficits [61,62]. 

After a TBI, an excessive immune response can harm the central nervous system, causing neurological damage and affecting long-term outcomes. Microglia and astrocytes, when activated, release inflammatory substances like TNF-α, interleukin-1 beta (IL-1β), and cyclooxygenase 2 (COX-2), mediated by nuclear factor-kappa B (NF-κB) [63]. Activated microglia release pro-inflammatory products that regulate astrocyte activation (astrogliosis) and glial scar formation. These processes hinder axonal repair and lead to neuronal cell death [64]. 

A study has shown that administering a NAD-dependent protein deacetylase Sirtuin-1 (SIRT1) activator, a histone deacetylase, can reduce NF-κB acetylation, leading to a decrease in neuroinflammation and the associated apoptotic pathway induced by TBIs [65]. A prolonged activation of NF-κB, a regulator of about 500 genes, many of which are involved in inflammation [66], can indeed lead to an overproduction of pro-inflammatory cytokines, exacerbating neuronal and axonal damage, disrupting the BBB, and increasing intracranial pressure. Annexin 5 effectively attenuated brain inflammation by inhibiting microglial activation and promoting their transition from a pro-inflammatory M1 to a reparative M2 state, thereby preventing ferroptosis and oxidative stress damage. In this case as well, the shifting was associated with the decrease in Iba1 and GFAP levels [67]. Shifting microglia from the M1 state to the M2 state has been found to reduce inflammation and promote tissue repair after a TBI [68]. This change is thought to be connected to the deactivation of the STAT3/NF-κB pathway. Inhibiting NF-κB could lower cytokine levels and affect apoptosis-related proteins, which are important for neuron preservation and recovery [69,70]. Apoptosis and necrosis are the primary cell death types post-TBI. An imbalance in pro- and anti-apoptotic proteins triggers cell death, mainly through the Caspase-3 pathway [71]. In TBI mouse models, intracranial injections of a trans-activator of transcription (TAT), a kinase inhibitor (KIR) compound, reduced reactive astrocytes and protected neurons by suppressing the Janus kinase 2 (JAK2)/STAT3 pathway, improving neurological function [72]. Moreover, abrocitinib, a JAK1 inhibitor, has been observed to prevent microglial transition by targeting the JAK/STAT/NF-κB pathway, suggesting it as a potential therapeutic approach [73]. 

Natural remedies like *Satureja khuzistanica Jamzad* essential oil, rich in carvacrol, have been effective in reducing acute edema and inflammatory responses post-TBI by partially inhibiting NF-κB signaling [74]. Another study demonstrated that papaverine provided neuroprotection by inhibiting receptor for advanced glycation end products (RAGE) and NF-κB signals, resulting in an anti-apoptotic and anti-inflammatory effect, potentially restraining microglial activation after TBI [75]. 

Moreover, there is growing evidence suggesting that post-TBI, elevated levels of NF-κB trigger the activation of the inflammasome complex, particularly the NLR family pyrin domain containing 3 (NLRP3). This activation process, involving the apoptosis-associated speck-like protein containing a Caspase-recruitment domain (ASC) and Caspase-1, leads to the release of the cytokines IL-1β and interleukin-18 (IL-18), which may be critical for the progression of a TBI [76]. Research by Cai L. et al. demonstrated that ACT001, an orphan drug, effectively reduced neuroinflammation and improved recovery in TBI models, primarily through inhibiting protein kinase B (AKT) phosphorylation, NF-κB activity, and NLRP3 inflammasome formation. Interestingly, the treatment with ACT001 also partially promoted certain anti-inflammatory microglial phenotype markers, such as Arg1, CD206, TGF-β, and IL-10 [77]. However, inhibiting the key modulators, such as NLRP3, Caspase-1, and gasdermin D (GSDMD), has shown potential in reducing neuronal death. The NLRP3/GSDMD signaling pathway could be implicated in neuropathological changes following a TBI, especially in the early stage. The NLRP3 inflammasome pathway mainly activated and cleaved GSDMD, leading to pyroptosis. The absence of GSDMD has been shown to attenuate neuroinflammation, the release of cytokines, both pro-inflammatory and anti-inflammatory, but further research is needed to explore the long-term effects of GSDMD on TBI-induced damage [78]. However, pyroptosis, an inflammation-driven form of cell death, is particularly prominent in neurons and microglia early post-TBI. Elevated expression of pyroptosis-associated proteins and messenger RNAs has been observed in animal models during the acute phase of TBIs [79]. 

Furthermore, the suppression of the phosphatidyl inositol 3-kinase (PI3K)/AKT/ mammalian target of the rapamycin (mTOR) and AKT/IκB kinase (IKK)/NF-κB pathways has been associated with the neuroprotective effects of urolithin A, which was found to reduce BBB disruption, edema, apoptosis, and improve neurological deficits [80]. Another research showed that the activation of the AKT signaling pathway in microglia, along with its downstream factors, cAMP response element-binding protein (CREB) and Brain-derived neurotrophic factor (BDNF), had a neuroprotective effect against TBIs, reducing neuronal damage and improving neuronal deficits [81]. Treatment with a metabotropic glutamate receptor 5 (mGluR5) positive allosteric modulator, called VU0360172, may activate AKT, inhibiting the Glycogen synthase kinase-3 beta (GSK-3β), which then increases CREB phosphorylation, affecting inflammation-related gene expression and microglial plasticity. It underscores the role of glutamate receptor signaling in mediating neuroinflammatory responses in TBIs [82]. Quinpirole, a medication classified as a dopamine D2 receptor (D2R) agonist, demonstrated its ability to mitigate various neuropathological processes through D2R/Akt/GSK-3β/IL-1β signaling within the ipsilateral cortex and striatum of an injured mouse brain. One potential effect of D2R stimulation is the reduction in the levels of GFAP and Iba1, which results in decreased glia activation and neuroinflammation [83]. 

#### 4.1.2. Antioxidant Strategies in Traumatic Brain Injury (TBI) Therapy

Recent studies have shed light on the complex interplay of various pathways in response to TBIs. Chronic microglia activation resulting from brain damage leads to the accumulation of ROS and upregulation of cytotoxic mediators such as TNFα, High Mobility Group Box 1 (HMGB1), and Inducible Nitric Oxide Synthase (iNOS). This cascade of events contributes to the overall neuroinflammatory response observed in TBIs. Central to counteracting this process is the activation of the nuclear factor erythroid 2–related factor 2 (Nrf2) pathway, which plays a pivotal role in triggering several antioxidant enzymes that aid in reducing oxidative damage and neuronal loss [84], thus suppressing neuroinflammation [85]. Nrf2, functioning as a transcription factor, maintains redox balance within the body and provides neuroprotection against TBI-induced oxidative stress in vivo [86,87]. 

Further research has highlighted the critical role of Nrf2 and Hemeoxygenase-1 (HO-1) in facilitating the effects of compounds like astaxanthin or hydrogen-rich saline solutions, which are known to reduce ROS production and prevent neuronal apoptosis [88,89]. This underscores the therapeutic potential of targeting the Nrf2/HO-1 pathway to protect against synaptic and cognitive dysfunction following a TBI [90].

In line with these findings, a study has demonstrated the effectiveness of atorvastatin, traditionally used for cholesterol management, in reversing the upregulation of endoplasmic reticulum (ER) stress proteins and reducing apoptosis, thereby improving neurological outcomes post-TBI, potentially through the activation of Nrf2/HO-1 pathway [91]. This highlights the efficacy of a combined therapy approach, which targets multiple pathological pathways, offering a comprehensive solution for the complex challenges of TBIs. Such an approach could lead to beneficial outcomes in both the short and long term. For example, a tri-combo therapy comprising apocynin, tert-butylhydroquinone, and salubrinal has shown promise in addressing oxidative stress, ER stress, and inflammation [92]. Continuous neuronal endoplasmic reticulum (ER) stress has been linked to neuroinflammation after a TBI, although the underlying mechanisms remain not fully understood, ER stress in mice could potentially shift the unfolded protein response (UPR) from a pro-survival to a pro-death state and lead neurons towards apoptosis. This is evidenced by the increased expression of ER stress markers like glucose-regulated protein-78 (GRP78), activating transcription factor 4 (ATF4), and C/EBP homologous protein (CHOP) following a TBI. In this context, naringenin has emerged as a neuroprotective agent by modulating UPR signaling, thereby influencing the ER stress-induced apoptotic pathway [93]. 

#### 4.1.3. Glutamate Modulators to Enhancing Neuronal Survival

Other studies have recently highlighted the significance of multifaceted approaches to enhance neuronal survival. In particular, Pleckstrin homology domain and leucine-rich repeat protein phosphatase (PHLPP) inhibitors, such as NSC74429, have shown promise in protecting against the damaging effects of oxidative stress triggered by hydrogen peroxide and excitotoxicity induced by glutamate [94]. Furthermore, hydrogen sulfide (H_2_S), a newly recognized gaseous neurotransmitter, has demonstrated neuroprotective effects by reducing oxidative stress associated with glutamate, potentially through the p53/glutaminase 2 pathway [95]. Excitotoxicity is considered an important secondary injury mechanism following a TBI, involving impaired neuronal calcium regulation and excessive glutamate release, resulting in dendrite damage. Glutamate plays a central role in TBIs but through different mechanisms, such as the modulation of glutamate receptors and the regulation of its transporters [96]. Research has shown that edonerpic maleate offered protection against TBI-related neuronal damage by modulating glutamate receptors subunits, such as GluR1, which helped in reducing calcium accumulation, neurotoxicity, and oxidative stress. Notably, an improvement in long-term neurological function was noted [97].

#### 4.1.4. Antiferroptotic Strategies Enhance Neurorepair in Traumatic Brain Injuries (TBIs)

Ferroptosis is a distinct form characterized by iron-dependent lipid peroxidation and the collapse of redox balance. In TBIs, the disrupted BBB permits excessive iron entry into the brain, enhancing ferroptosis due to diminished glutathione peroxidase 4 (GPX4) enzyme activity, a key defense against lipid peroxidation. Currently, there is no specific treatment for ferroptosis in clinical practice, but targeting this pathway shows promise for neuroprotection and recovery post-TBI. Ruxolitinib, a JAK inhibitor, has been noted for its potential in reducing iron deposition and tissue loss, indicating its potential beyond existing [98]. 

Huang et al. reported that TBIs may impair GPX4 activity through protein modification, underscoring the need for interventions to reverse trauma-induced molecular alterations. The authors demonstrated that administering polydatin after a TBI effectively preserved neuronal viability by restoring GPX4 activity and inhibiting ferroptosis, thus reducing brain damage and functional deficits [99]. Additionally, Netrin-1, involved in nerve regeneration, may facilitate the upregulation of GPX4 by promoting Nrf2 transcription and nuclear translocation [100]. Nrf2 knockout mice showed increased neuronal damage and neurological impairments compared to wild-type mice, along with increased iron accumulation induced by TBIs, which exacerbated lipid peroxidation and ferroptosis in neurons. The anti-ferroptotic role of Nrf2 after a TBI might be linked to its ability to modulate iron metabolism by upregulating ferritin components and the sodium-independent cystine–glutamate antiporter, system xc-(xCT)/GPX4/Ferroptosis suppressor protein 1 (FSP1) pathway [101]. Ferritin, comprising light chain-ferritin (FTL) and heavy-chain ferritin (FTH), is crucial for converting and storing iron, thus mitigating TBI-induced neurological impairments by reducing ROS production. Studies have identified melatonin as an inhibitor of neuronal ferroptosis induced by TBIs, acting through FTH. This hypothesis was supported by the elevated levels of ROS and iron found in FTH knockout mice after brain trauma [102]. Melatonin offers multiple protective benefits in TBI management, including reduced astrocytic reactivity and neuronal apoptosis and enhanced cognitive functions [103]. Melatonin also ameliorated mitochondrial dysfunctions in TBIs, as evidenced by restored mitochondrial membrane potential and oxidative phosphorylation, along with reduced cytochrome c release in treated rats [104]. 

Additionally, post-TBI, FTH levels increase, possibly as a response to injury. H_2_S has emerged as a prospective therapeutic agent, with anti-ferroptotic properties, enhancing brain function recovery following a TBI. Notably, the Wnt signaling pathway was significantly reduced after controlled cortical impact in mice, and its activation might be crucial in the neuroprotective effects associated with H_2_S in the context of TBIs [105]. Anacardic acid has also shown promise in alleviating neurological and behavioral cognitive impairments from TBIs via the dual effect of anti-ferroptosis and anti-inflammation [106]. 

#### 4.1.5. Multifaceted Approaches to Traumatic Brain Injury (TBI) Treatment from Animal Models to Clinical Trials 

Research with animal TBI models indicates that focusing on secondary injury mechanisms could yield treatments that significantly improve cognitive functions [107,108]. These findings provide a fresh perspective on TBI treatment, underscoring the need for a multifaceted approach to tackle different facets of neuronal damage and recovery. Inhibiting processes like neuroinflammation and oxidative stress can aid in preventing neuronal loss and reducing brain tissue damage, thus lowering the risk of neurodegeneration [109,110]. 

Although there have been advancements, the FDA has yet to approve specific TBI therapies, and few drugs have been clinically adopted. Repurposing drugs like levetiracetam, which reduces post-traumatic seizures in TBI patients [111,112], is a quick treatment option. Yet, its role in overall brain damage reduction is limited, as it does not prevent cortical damage associated with seizures. Due to the diverse nature of TBIs and differing vulnerabilities of brain structures, an integrated, multidisciplinary approach to evaluating therapies is crucial. Neuroprotective effects observed in one model may not apply to another [113]. Additionally, the use of preventive therapies like Immunocal is increasingly recognized for patients at high risk of repeated TBIs, particularly when combined with treatments like mitochondrial protectants or anti-glutaminergic agents [114].

A combined approach using in silico analysis, in vitro studies, animal models, and clinical trials is essential for effectively evaluating treatment efficacy. The effective management of secondary injuries supports neuroprotection and the potential for neural function recovery, which could enhance the prognosis for TBIs. The potential synergy of specific compounds in modulating oxidative stress, ER stress, and inflammation presents a promising strategy for treating TBIs. Moreover, the decreased expression of the biomarkers GFAP and Iba1 may be associated with an anti-inflammatory phenotype of glial cells, which is linked with improved neurological function. However, the studies highlight the need for further research to identify the optimal timing and dosage for these therapies and explore more deeply their neuroprotective and anticoagulant properties, indicating a significant potential for clinical translation in TBI treatment.

**Figure 2:**
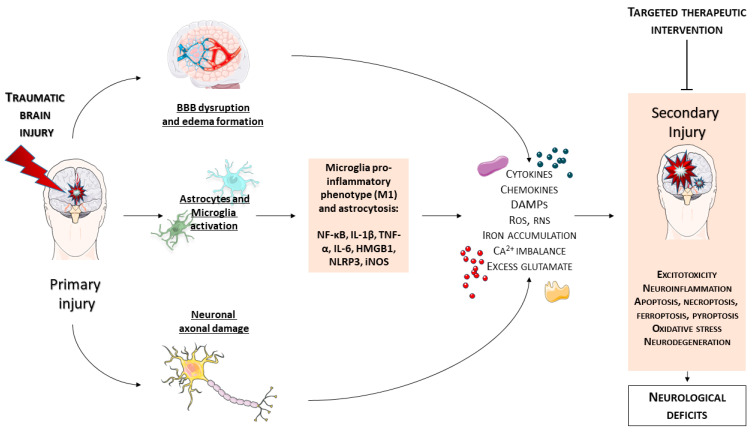
Pathological changes associated with TBIs begin with a direct physical injury, known as a primary injury, which disrupts the BBB, damages axons, and activates glial cells. Subsequently, DAMPs released from injured cells can overactivate immune cells, leading to an increased production of pro-inflammatory factors that amplify the inflammatory response, thereby worsening the injury. Additionally, iron accumulation exacerbates oxidative stress and facilitates ferroptosis. At the same time, the excessive neuronal release of glutamate post-TBI lead to hyper stimulate glutamate receptors, increasing the influx of calcium and resulting in excitotoxicity. These interconnected processes significantly contribute to the secondary injury phase, potentially contributing to the cellular death in the brain and to neurological impairments. This image was created using the image bank of Servier Medical Art (Available online: http://smart.servier.com/; accessed on 30 December 2023) licensed under a Creative Commons Attribution 3.0 Unported License (available online: https://creativecommons.org/licenses/by/3.0/, accessed on 30 December 2023). BBB: blood–brain barrier; Ca^2+^: calcium; DAMP: damage-associated molecular pattern; HMGB1: High Mobility Group Box 1; IL-1β: interleukin-1 beta; IL-6: interleukin-6; iNOS: Inducible Nitric Oxide Synthase; NF-κB: nuclear factor-kappa B; NLRP3: NLR family pyrin domain containing 3; RNS: Reactive Nitrogen Species; ROS: reactive oxygen species; TBI: traumatic brain injury; and TNF-α: tumor necrosis factor-alpha.

In conclusion, as summarized in Table 2, several treatments targeting inflammation and immune cell activation show promise in reducing TBI-related damage. Key findings include the protective effects of natural remedies like essential oils and compounds targeting the NF-κB pathway. These studies also emphasize the importance of regulating pathways like NLRP3 inflammasome, Nrf2/HO-1, ER stress, and AKT/GSK-3β for neuroprotection. Additionally, recent research highlights the potential of targeting ferroptosis, a distinct iron-dependent form of cell death, to prevent the exacerbation of neuronal damage and promote recovery post-TBI (Figure 2). The complexity of TBI necessitates a multifaceted approach, combining pharmacological interventions with natural remedies to address various aspects of brain injury and repair.

### 4.2. Regenerative Medicine

Mesenchymal stem cells (MSCs) are multipotent progenitor cells that are well-suited for cell-based therapy [115]. These cells possess the unique ability to regulate the immune system, characterized by their secretion of a range of factors, potential for differentiation into diverse cell types, and inherent immunomodulatory properties. Specifically, when exposed to inflammatory agents like TNF-α and IL-1, MSCs become activated and exert immunosuppressive effects [116]. MSCs function by releasing a variety of soluble factors, such as growth factors, cytokines, chemokines, EVs, anti-inflammatory molecules, and neurotrophic factors. These soluble factors enhance cell survival and attenuate neuronal apoptosis [117,118], regulate inflammation [119,120], and support angiogenesis [121,122], neurogenesis, and synaptic plasticity [123].

Emerging studies have unveiled that MSCs’ therapeutic efficacy is closely tied to exosomes, minute vesicles ranging from 30 to 150 nanometers. These exosomes encapsulate lipids, proteins, non-coding RNAs, and messenger RNAs [124]. They assume a critical role in facilitating cellular communication and exhibit the capability to breach the BBB [125]. This suggests their promising application in diagnosing and managing central nervous system conditions, including TBIs [126,127].

Tang, L. et al. explored the therapeutic potential of exosomes derived from adipose-derived MSCs (AD-MSCs) in the context of TBIs. This study employed both in vitro and in vivo models to assess their ability to mitigate inflammation and promote neurological recovery following a TBI. In the in vitro model, microglial cells exposed to lipopolysaccharide (100 ng/mL) were treated with AD-MSC-derived exosomes (5 or 10 μg/mL), demonstrating a reduction in inflammation. In the in vivo study, rats received intravenous injections of these exosomes (200 μg) after TBI induction, resulting in a decrease in secondary inflammation. This suggests that AD-MSC-derived exosomes hold promise as a potential treatment for inflammation and neurological damage post-TBI [128].

Research has demonstrated that exosomes derived from human umbilical cord MSCs (HucMSCs) improve neurological function, reduce brain swelling, and decrease lesion size after a TBI. Exosomes (100, 150, 200, 250, and 300 μg/mL) were administered via the tail vein 30 min after a TBI. The optimal concentration for protection is 200 μg/mL. They inhibit inhibiting multiple cell death pathways, including apoptosis, pyroptosis, and ferroptosis. Additionally, they promote mitophagy through the PINK1/Parkin pathway, a critical mechanism for safeguarding the brain from damage. Exosome treatment holds promise as a TBI therapy [129]. Exosomes from HucMSCs provide neuroprotection against TBIs by activating the lncRNA TUBB6/Nrf2 pathway, reducing inflammation and ferroptosis. Knocking down TUBB6 partially diminishes their neuroprotective effect, highlighting their importance [130]. Further research is required to determine whether their non-coding RNAs contribute to this effect and if adjusting the timing of exosome administration can enhance their neuroprotection against TBIs.

MSC-derived exosomes are recognized for their immunomodulatory functions and their role in tissue regeneration. In contrast, exosomes derived from astrocytes play a critical role in reducing oxidative stress and apoptosis in neuronal cells after brain injuries [131]. Both types of exosomes underscore their therapeutic potential in neurology, encouraging the investigation of various cellular sources to improve treatments for TBIs. In a study by Zhang et al., astrocyte-derived exosomes were used to treat TBIs in animal models. These exosomes, administered 30 min after the TBI at a dose of 100 μg intravenously, improved neurobehavioral deficits, cognitive impairment, and brain edema. Their effectiveness was attributed to reduced oxidative stress and neuronal apoptosis through the Nrf2/HO-1 pathway, showing their potential for TBI treatment [132].

**Figure 3:**
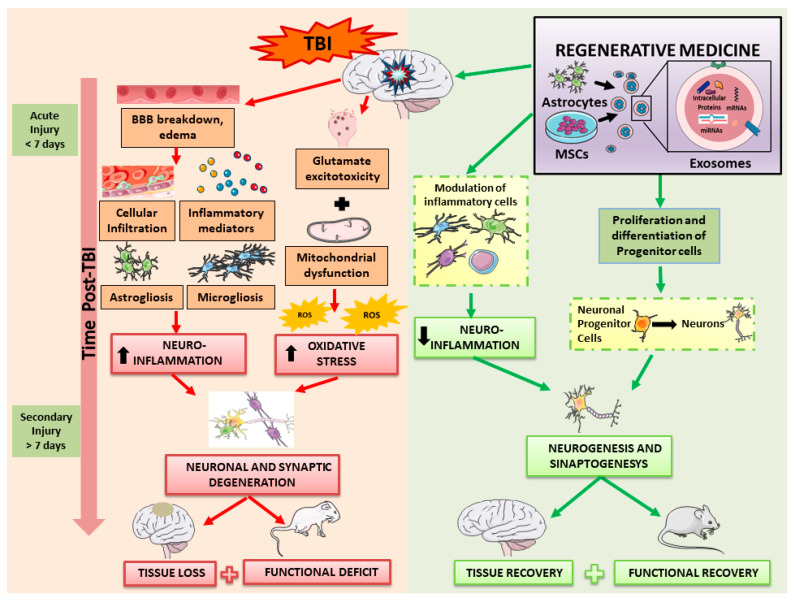
Regenerative medicine. MSCs secrete biologically active molecules that can influence various molecular pathways. These treatments support the survival and proliferation of regional cells by secreting chemokines and growth factors. A significant aspect of their function is the reduction in edema and inflammation caused by injury, enhancing the secretion of anti-inflammatory cytokines while simultaneously reducing the secretion of pro-inflammatory cytokines. These multifaceted approaches highlight their significant impact in facilitating recovery from TBI, highlighting their ability to support tissue repair, mitigate inflammation, and promote neural regeneration. The image was created using the image bank of Servier Medical Art (available online: http://smart.servier.com/; accessed on 30 December 2023) licensed under a Creative Commons Attribution 3.0 Unported License (available online: https://creativecommons.org/licenses/by/3.0/; accessed on 15 December 2023). BBB: blood–brain barrier; MSCs: mesenchymal stem cells; ROS: reactive oxygen species; and TBI: traumatic brain injury.

In summary, research on exosomes derived from both MSCs and astrocytes has demonstrated significant therapeutic potential in the treatment of traumatic TBIs (Figure 3). While MSC-derived exosomes focus on immune modulation and tissue regeneration, those from astrocytes reduce oxidative stress and apoptosis in neuronal cells. These findings open new perspectives in neurology and suggest a wide range of potential treatments for TBIs, highlighting the need for further research to optimize therapeutic efficacy in different disease contexts.

## 5. Challenges and Future Prospects

TBIs remain a complex medical challenge, with an urgent need for effective treatments within the healthcare system and for patients. The brain’s response to trauma, encompassing processes like inflammation, neurogenesis, and plasticity, often fails to halt the progression of damage. In this context, the use of cell death suppressors in combination has emerged as a potential therapeutic strategy for managing TBIs. Despite extensive research, an effective and approved treatment for TBI is still not available, largely due to the diverse responses observed in humans. The heterogeneity of pathological alterations significantly hinders the successful clinical application of promising pre-clinical therapies. Advances in biomarker discovery and neuroimaging are expected to aid in early diagnosis, monitoring of TBI progression, and assessing treatment efficacy. Currently, TBI management primarily focuses on symptomatic relief and supportive care, lacking established protocols for long-term complications [9]. Furthermore, the detailed process by which TBIs lead to chronic neurological impairment remains largely undefined.

There is an increasing emphasis on developing targeted therapies that address specific pathways, which play a significant role in nerve damage following a TBI. Understanding the cellular interactions involved in a TBI is crucial. Important aspects include validating the mechanisms of these therapies in animal models, ensuring they are effectively targeted by the treatments, confirming their relevance in human TBI, determining the drug’s ability to penetrate the brain effectively, and verifying their safety and tolerability in cases of human head injury [133].

Integrating various in vitro and in vivo models could increase the chances of obtaining reliable and promising results, minimizing the risk of false positive neuroprotective effects from drugs and treatments. However, in vitro TBI models also have limitations, including the potential for cell damage during sampling [134].

However, many therapies that appear promising in animal and cell-based laboratory models fail in clinical trials, highlighting the gap between pre-clinical and clinical research. This gap underlines the importance of pre-clinical studies employing models that more accurately mimic human TBI, thereby enhancing the chance of successful clinical translation. While animal models have physiological similarities with humans, significant differences in brain structure and function pose challenges in accurately modeling human TBI. Additionally, many studies with animal models of TBI often omit specifying the injury’s severity. Further investigation is also necessary to fully understand how age, sex, and species affect TBI outcomes [135].

## 6. Conclusions

The most accredited biomarkers for prognostic indicators in TBIs currently include NF-L, GFAP, Tau, and Brain-derived Tau. These biomarkers have shown significant reliability in reflecting the severity and potential outcomes of TBIs. Regarding recent pharmacological advances, the most promising strategies for TBIs are aimed at targeting neuroinflammation, oxidative stress, and apoptotic pathways. Approaches that emphasize anti-inflammatory immune modulation and inhibition of detrimental pathways, such as NF-κB and inflammasomes, demonstrate potential for enhancing both clinical and neurological outcomes. Several therapeutic treatments have shown promise in improving neurological function by modulating glial activity, as evidenced by the decreased levels of biomarkers such as GFAP. Additionally, antioxidant molecules, particularly melatonin and astaxanthin, have demonstrated potential for mitigating TBI by addressing oxidative stress and ferroptosis. Overall, targeting multiple pathways involved in secondary injuries may promote neural recovery by preventing damage progression and neurodegeneration. In this evolving landscape, emerging research on exosomes derived from both MSCs and astrocytes presents a promising frontier in neurology. These exosomes have the potential to revolutionize TBI treatment by focusing on immune modulation, tissue regeneration, and reducing oxidative stress and neuronal apoptosis. However, further investigations are needed to identify the ideal timing and quantity of these therapies and to fully understand how they can be used effectively in clinical TBI management.

## Figures and Tables

**Table 1:** This table summarizes the types of pre-clinical and clinical studies, their experimental designs, the biomarkers investigated, and the primary outcomes or results discovered. These findings reveal significant correlations between these biomarkers and various Traumatic Brain Injury (TBI) outcomes, including symptom severity and neurodegenerative processes, highlighting their potential in diagnosing, prognosing, and understanding TBIs.

Condition	Type of Study	Experimental Design	Biomarkers	Results	Ref.
**Preclinical studies**					
MTBI induced by low-intensity blast	In vivo study	Two-month-old male C57BL/6J mice were randomly assigned to a control group and a group of animals exposed to a low-intensity blast that induced mTBIs. To induce mTBIs, the animals are placed in metal containers in a prone position, three meters away from the detonation of a 350 gr C4 explosive. NF-L and GFAP levels were measured in the brain tissues and plasma of mice post-trauma exposure.	GFAP and NF-L	Low-intensity blast-induced mTBI increased GFAP and NF-L levels in the brain tissues and plasma of the mice. These increases may indicate damage to the BBB and potentially predict the extent of neuronal and glial damage.	[25]
Repeated mTBI	In vivo study	Sixty-four male Long–Evans rats were divided into a control group that underwent sham injuries and a group that underwent four repeated mTBIs. Serum NF-L quantification was performed, as well as mass spectrometry analysis of the hippocampal proteome to further evaluate the effects of mTBIs at 7 days and 3.5 months post-injury.	NF-L	An acute, but not chronic, increase in serum NF-L levels was observed. Furthermore, significant changes were detected in 26 proteins at 7 days and in 72 proteins at 3.5 months after mTBI.	[26]
TBI	In vivo study	A large cohort of Yucatan swine (*n* = 21) exposed to severe TBI and 68 Yucatan swine exposed to severe TBI + hemorrhagic shock (40% blood loss) or to sham trauma procedures (*n* = 12) were measured the circulating levels of NFL, GFAP, and UCH-L1.	GFAP, UCH-L1, and NF-L	GFAP, NF-L, and UCH-L1 are sensitive to multiple forms of trauma	[27]
Clinical trials					
**Biomarkers of Neuronal, Glial, and Axonal Damage in TBI**					
Mild, moderate, and severe TBI	An observational clinical study (NCT02119182)	Participants (aged 17–90 years) from the Transforming Research and Clinical Knowledge in TBI (TRACK-TBI) study were included in the study, and plasma samples were collected on the day of injury to assess the value prognosis of GFAP and UCH-L1 proteins.	GFAP and UCH-L1	The plasma levels of the GFAP and UCH-L1 proteins, measured immediately after a TBI, demonstrate a good ability to predict the long-term outcomes of patients. These values suggest a relatively high capacity of these biomarkers to predict these specific outcomes.	[30]
Mild and moderate/severe TBI	A prospective clinical study	This study analyzed 109 TBI patients recruited within 6 h of injury. A hyperacute subgroup of 20 patients was analyzed separately to identify early acute biomarker levels.	GFAP, UCH-L1, and S100B	GFAP and UCH-L1, but not S100B, levels were significantly higher in patients with intracranial abnormalities visible on CT (CT-positive) compared to CT-negative patients. This study supports the clinical utility of GFAP and UCH-L1 as biomarkers in TBI and differentiating injury severity.	[33]
Patients with mild or severe TBI within 24 h after a lesion	A case–control study	This study included 224 participants, divided into four groups based on injury severity: no injury (*n* = 77), physical injury (*n* = 37), uncomplicated mTBI (*n* = 55), and more severe TBI (*n* = 55). A subgroup (*n* = 87) completed follow-up cognitive assessments. Participants underwent a blood draw and neuropsychological evaluation within one year of their injury.	GFAP, UCH-L1, Tau, and NF-L	In the severe TBI group, a baseline level of UCH-L1 and GFAP predicted change in immediate and delayed memory over time. Instead, in the mTBI group, higher baseline Tau levels predicted greater negative change in perceptual reasoning and executive function, while higher baseline levels of NF-L predicted greater negative change in perceptual reasoning.	[34]
All severities (mild, moderate, and severe) of TBI	A prospective study (NCT02210221)	Serum levels of six biomarkers were measured in 2869 patients with TBIs of varying severity within 24 h of injury.	GFAP, UCH-L1, Tau, S100B, and NF-L	These biomarkers increased in relation to the severity and volume of brain lesions.	[35]
mTBI	A prospective study (NCT02988102)	Pediatric patients (between 7 and 16 years) with mTBIs were enrolled, and serum levels of S100B to 3 h from injury were measured.	S100B	Higher levels of S100B protein in serum were associated with the presence of post-concussion syndrome and with the severity, highlighting that it is a valid biomarker for identifying pediatric patients at risk of developing post-concussion syndrome after a mTBI.	[36]
Mild to severe TBI	A prospective study	460 children aged 0 to 21 years, with head injuries of various severities were included. Within 24 h of injury, they were evaluated to determine the effectiveness of plasma osteopontin and S100B in predicting mortality and functional outcomes after 6 months.	S100B and osteopontin	Osteopontin and S100B correlated with injury severity, with S100B showing larger usefulness in predicting mortality and 6-month outcomes.	[37]
Mild to severe TBI	A prospective observational stud	87 children, aged 1 to 17 years, who suffered from mild to severe TBI were enrolled. Within 24 h of injury, this study explored whether serum biomarker concentrations could predict persistent fatigue 12 months post-injury.	IL-8	IL-8, an inflammation marker secreted by various cells in response to brain injury, proved useful in predicting persistent fatigue 12 months after the injury.	[38]
Sub-concussive TBI	A case–control study	Men’s college football players were divided based on their playing position (fast positions and slow positions). Biomarker levels were performed before and after the playing season.	GFAP, UCH-L1, Tau, and NF-L	During the football season, the blood levels of two markers, GFAP and NF-L, went up, even though few players had concussions. Also, after the season, Tau and NF-L were higher in fast-playing positions, like quarterbacks and receivers, and lower in slower positions, like linemen.	[39]
Sub-concussive TBI	A large multi-centric study	496 male professional rugby players were enrolled in this protocol to determine their baseline serum biomarker concentrations and assess the effect of rugby on blood biomarkers over a season. A total of 45 sport-related concussion cases were reported for 42 players whose blood collection was at 36 hours after injury.	S100B, NSE, SBDP, UCHL-1, GFAP, NF-L and Tau.	There were no significant differences in biomarker concentrations measured 36 h after sport-related concussion between players with non-resolving and resolving damage. Only S100B and NF-L showed significant differences between these two groups, with S100B demonstrating a better performance than the other biomarkers.	[40]
mTBI	A multicenter prospective case–control study	1085 United States military cadets were enrolled in this study, of whom *n* = 67 sustained concussions, while *n* = 36. They participated in the same combat training exercises but did not suffer any trauma. Blood samples were collected: post-acute injury (<6 h); 24 to 48 h after injury; asymptomatic post-injury; and 7 days after return to activity.	GFAP, UCH-L1, NF-L, and Tau	Significant increase in GFAP and UCH-L1 levels immediately after trauma.	[41]
Combined mild, moderate, severe, and penetrating complicated TBI	A 15-year longitudinal clinical study	84 United States military personnel and veterans were prospectively enrolled in the 15-year longitudinal TBI study and were divided into mild uncomplicated TBI (*n* = 28), combined mild, moderate, severe, and penetrating complicated TBI (*n* = 29), and uninjured subjects (*n* = 27) to evaluate the relationship between Tau, NF-L, GFAP, and UCHL-1 concentrations with neurobehavioral symptoms within 12 months of injury, and then again at 2 or more years post-injury.	GFAP, UCH-L1, NF-L, and Tau	Tau, NFL, GFAP, and UCHL-1 have been associated with the deterioration of neurobehavioral symptoms within 12 months of injury.	[42]
Repetitive head trauma	A longitudinal clinical study	This study included 140 active boxers, 211 active MMA fighters, 69 retired boxers, and 52 control participants and analyzed baseline levels of the biomarkers NF-L and GFAP. The correlation between increased GFAP levels and deterioration of cognitive performance in active fighters has also been studied.	GFAP and NF-L	NF-L levels were higher in active boxers compared to Mixed Martial Arts fighters. In comparison, GFAP levels were higher in retired boxers with a correlation to decreased volumes of multiple brain structures and cognitive decline over time.	[27]
**Biomarkers of Synaptic Damage and Dysfunction**					
Polytraumatic TBI	A longitudinal clinical study	Patients with severe injury (*n* = 32) and healthy subjects (*n* = 13) were enrolled in the trial to analyze plasma NF-L, beta-synuclein, and GFAP levels at 0 h, 24 h, 5 days, and 10 days after injury.	NF-L, beta-synuclein, and GFAP	Beta-synuclein and GFAP reached an elevated level early after injury, while NFL increased gradually over the course of 10 days.	[43]
Sport-related concussion	A prospective multicenter case–control study	Collection of blood samples within 48 h of injury to identify protein abnormalities in 161 concussed (*n* = 140) and non-concussed (*n* = 21) athletes.	EPABA41 and SNCA	Among the 1305 proteins identified, 319 were upregulated and 19 downregulated in concussed athletes compared to non-concussed athletes. Specifically, 6 h after injury, EPB41 and SNCA emerged as biomarkers useful for diagnosing concussion in athletes.	[44]
**EVs as Biomarkers with NFL, Tau, and GFAP in Focus**					
Chronic mTBI	A multicenter prospective longitudinal study	A cohort of military service members and veterans (*n* = 144) with chronic mTBI were divided according to their mTBI and severity of post-traumatic stress disorder symptoms, and levels of EV proteins and microRNAs measured in the peripheral blood were measured.	EVs, NF-L, Tau, Aβ42, Aβ40, IL-10, IL-6, and TNF-α	Significant differences in NFL in EV and plasma levels were detected between subjects with mTBI and post-traumatic stress disorder symptoms groups.	[45]
mTBI and complex TBI	A 15-year longitudinal clinical study	218 service members and veterans were divided into uncomplicated mTBIs (*n* = 107); complicated mild, moderate, or severe TBIs (*n* = 66); or orthopedic injury without TBI (*n* = 45) and were measured the levels of NFL and GFAP in EVs isolated from blood within the first year after injury.	NFL and GFAP in EVs	NFL in EVs isolated from blood higher in complex TBI group compared to mTBI with a dose–response relationship between TBI severity and NFL concentrations.	[46]
Moderate and severe TBI	A prospective, observational clinical study	Subjects (*n* = 114) with and without a TBI were enrolled to analyze their total EV levels of proteins using single-molecule array technology.	GFAP, UCH-L1, NF-L, and Tau in EVs	Protein levels (GFAP, UCH--, NF-L, and Tau) in EVs increased with the severity of TBI, highlighting a correlation between the levels of biomarkers in plasma and those encapsulated in EVs.	[47]
mTBI	A observational clinical study	mTBI subjects and healthy subjects were enrolled to obtain an aliquot of plasma to isolate brain-derived EVs based on their expression of GluR2+ using an innovative nanofluidic platform, TENPO. Lysates from GluR2+ EVs and a second aliquot of plasma were used to analyze TBI biomarkers by ultrasensitive digital ELISA.	GFAP, UCH-L1, NF-L, Tau, TNF-α, IL-10, and IL-6 in GluR2+ EVs and plasma	GFAP and IL-6 were significantly elevated in both plasma and GluR2+ EVs in mTBI. Plasma levels of NF-L, Tau, and UCHL1 were all significantly elevated in the mTBI group, while the levels of these proteins in GluR2+ EVs remained unchanged after mTBI. TNF-α and IL-10 were significantly elevated in plasma in the mTBI group compared to the control group; on the contrary, none of these cytokines were significantly altered in the EV GluR2+ compartment after mTBI.	[48]
**Novel Selective Blood Axonal Injury Biomarkers**					
Mild, moderate, and severe TBI	A prospective cohort clinical study	39 patients with severe TBIs were enrolled to evaluate the level of serum Brain derived-Tau on days 0, 7, and 365 after damage.	Brain derived-Tau	Brain-derived Tau could be a valid biomarker to discriminate the severity of TBI and monitor the clinical outcomes relating to acute neuronal injuries.	[49]

Aβ: amyloid beta; ELISA: enzyme-linked immunosorbent assay; EPAB41: erythrocyte membrane protein band 4.1; EVs: extracellular vesicles; GFAP: glial fibrillary acidic protein; GluR2+: glutamate ionotropic receptor AMPA type subunit 2; IL-10: interleukin-10; IL-6: interleukin-6; mTBI: mild TBI; NF-L: neurofilament light chain polypeptide; NSE: neuron-specific enolase; S100B: sport-related concussion calcium-binding protein B; SBDP: spectrin breakdown product; SNCA: alpha-synuclein; TBI: traumatic brain injury; TENPO: Track Etched Magnetic Nanopore (TENPO); TNF-α: tumor necrosis factor-alpha; UCH-L1: ubiquitin C-terminal hydrolase-L1; and VEGF: vascular endothelial growth factor.

**Table 2:** An overview of recent pre-clinical research focused on neuroprotection following a Traumatic Brain Injury (TBI). Various treatments have been effective in improving brain tissue and mitigating post-traumatic effects under different trauma conditions. Promising treatments can target molecular mechanisms in signaling pathways related to secondary injury. Substances utilized in TBI models are categorized by therapeutic modality.

Condition	Treatment	Type of Study	Experimental Design	Molecular Mechanism Involved in Secondary Injury	Results	Ref.
**Targeting multiple pathways for neuroprotection in preclinical TBI studies**						
rmTBI	ALC	In vivo	Male C57BL/6 mice were divided into sham, rmTBI, and ALC + rmTBI groups. Mice were euthanized at either 48 h or 90 days after impact. ALC was administered at 600 mg/kg/day in saline, while the sham and rmTBI groups only received saline.	AIF1, GFAP, TNF, CCL11, GRIA1, TDP-43, and MAPT	Treatment with ALC showed neuroprotective effects at both early and chronic stages post-TBI, helping to reduce cognitive impairments caused by secondary injury.	[55]
CCI method	AST-004	In vivo	Male and female C57BL/6 J mice were divided into sham, TBI+vehicle, and TBI + AST-004 groups. AST-004 was i.p. injected at 0.22 mg/kg 30 min after injury. The duration of the experiments was 8 days.	GFAP and Iba1	AST-004, as a novel A3R agonist, reduced the astrogliosis biomarkers, while increased astrocyte energy production and enhanced their neuroprotective efficacy after brain injury. Thus, AST-004 reduced cell death and BBB disruption, improving long-term cognitive impairments post TBI. Female mice did not exhibit memory loss at 24 and 48 h after training.	[57]
TBI	Tibolone	In silico	The experimental design involves compiling gene lists from databases, analyzing gene functions and pathways, conducting a meta-analysis of biological terms, creating a protein–protein interaction network, and performing statistical analyses to explore the impact of tibolone and its metabolites on genes related to TBIs.	Estrogen receptors α and β, androgen receptors, and progesterone receptors	Tibolone metabolites α, β, and Δ, showed potential as TBI therapies due to their ability to activate biological processes known for their anti-inflammatory and antioxidant activity.	[58]
**Anti-inflammatory Agents and Immune Modulators in TBI**						
	JM-20	In vivo	Adult male Wistar rats were divided into four groups: (1) control; (2) JM-20 (8 mg/kg); (3) TBI + vehicle; and (4) TBI + JM-20. The treatment, either JM-20 or vehicle, was administered orally 1 h after the TBI. JM-20 was prepared in a 0.05% carboxymethylcellulose solution just before use.	TNF-α, IL-1β, BDNF, GDNF, and NGF	JM-20 treatment after mTBI improved behavioral performance, enhanced short-term memory, reduced brain edema, and decreased astrocyte reactivity and microglial activation. It also regulated pro-inflammatory cytokine release, suggesting its neuroprotective effectiveness in TBI contexts.	[61]
LPS exposure; CCI model	Fluvoxamine	In vitro and in vivo	BV2 cells, with or without LPS (1 μg/mL) stimulation, were treated with 10 μM of fluvoxamine. Male C57BL/6 mice were divided into sham, sham + fluvoxamine, TBI + vehicle (0.5% DMSO), TBI + fluvoxamine, and TBI + fluvoxamine + BD-1047. Fluvoxamine was administered i.p. at dose 10 mg/kg/day.	GFAP, Iba1, IKK, IκB, NF-κB, iNOS, Bcl-2, Bax, Caspase-9, and Caspase-3	GFAP and Iba1 levels were increased after trauma, but their levels decreased after treatment. Fluvoxamine treatment induced a phenotypic transformation of microglia/macrophages from a pro-inflammatory M1 phenotype to an anti-inflammatory M2 phenotype in both in vivo and in vitro experiments.	[62]
Cortical stab wound injury	Lupeol	In vivo	C57BL/6 mice were divided into four groups: control, which vehicle (0.5 mL saline/day); TBI; TBI with lupeol treatment (50 mg/kg/day/p.o.); and lupeol-alone treatment (50 mg/kg/day/p.o.). The duration of the experiment was 7 days.	GFAP, Iba1, NF-κB, TNF-α, COX2, IL-1β, Caspase-3, Cyto-C, Bcl-2, and Bax	Lupeol treatment in TBI mice decreased GFAP and Iba1 levels, resulting in reduced glial cell activation and pro-inflammatory molecules and lower oxidative stress markers such as ROS and lipid peroxidation. Instead, Nrf2/HO-1 expression was increased after treatment. Additionally, it reduces apoptotic molecules leading to improved memory and learning.	[63]
CCI model	SIRT1 activator	In vivo	Male Sprague Dawley rats were divided into sham, TBI, TBI + vehicle (0.9% saline + 3% DMSO), TBI + SIRT1 activator (5 mg/kg), and TBI + SIRT1 inhibitor (2 mmol/L, 30 μL/kg). SIrt1 activator was administered 30 min after TBI.	Bcl-2, BAX, SIRT1, NF-κB, TNF-α, and IL-6	Activation of SIRT1 reduced cerebral edema, neuronal apoptosis, and neurofunctional deficits after the TBI. Using a SIRT1 activator decreased NF-κB acetylation, suggesting SIRT1’s neuroprotective role against neuroinflammation-induced apoptosis.	[65]
FPI model	Recombinant human annexin 5	In vivo	Adult male C57BL/6 mice were divided into sham, TBI and TBI + annexin 5 groups. Annexin was administered at 50 µg/kg via a tail vein injection, while the sham and TBI groups received sterile saline.	GFAP, Iba1, HMGB1, NF-κB, Nrf2, HO-1, COX2, IL-1β, IL-6, IL-10, FTH, GSH, and GPX4	Annexin 5 showed neuroprotective effects against the TBI by improving cognitive and motor functions, reducing cerebral edema, lesion volume, oxidative stress, and ferroptosis. It inhibited neuronal apoptosis, neuroinflammation, and the HMGB1/NF-κB pathway while also enhancing the Nrf2/HO-1 antioxidant pathway. Additionally, treatment reduced GFAP and Iba1 levels.	[67]
CCI model	Maraviroc	In vivo	Male C57BL/6 mice were divided into sham, TBI + vehicle (5% DMSO, 40% polyethylene glycol 300, and 5% tween), amd TBI + maraviroc (20 mg/kg). Vehicle or maraviroc were administered via i.p. injection 1 h after injury.	GFAP, Iba1, HMGB1, NF-κB, NLRP3, Caspase-1, ASC, IL-1β, Caspase-3, and Bax	Maraviroc regulated the polarization of astrocytes and microglia, reducing GFAP and Iba1 levels. In addition, it mitigated neuroinflammation and neuronal damage post-TBI by modulating the key molecular pathways and cell types involved in the inflammatory response.	[68]
LPS and OGD/R models; CCI model	Parthenolide	In vitro and in vivo	BV2 and primary microglia were exposed to LPS (1 μg/mL), while HT22 cells were subjected to OGD/R model. Treatment with parthenolide was performed at 0.5–5 μM for 24 h. C57BL/6 mice were divided into sham, parthenolide (1 mg/kg), TBI, and TBI+ parthenolide.	STAT3, NF-κB, NLRP1, NLRP3, NLRC4, Caspase-1, Caspase-3, iNOS, GSH, SOD, COX2, Bax, Bcl-2, IL-1β, IL-6, TNF-α, IL-4, and IL-10	Parthenolide inhibited LPS-induced inflammation, oxidative stress, and apoptosis in vitro by blocking the STAT3/NF-κB pathway. The data confirmed that the treatment also suppressed inflammation by inhibiting microglia M1 activation. Additionally, in the TBI groups, the treatment improved brain damage, reduced neuronal loss, and enhanced learning and memory.	[69]
CCE treatment; severe CCI model	BAY61-3606	In vitro and in vivo	Primary microglia and astrocytes were extracted from neonatal mice induced with a TBI. Microglia were divided into control (10% DMSO), CCE (100 µL/mL), CCE+BAY (2 µM), and BAY groups. Male C57BL/10ScNJ mice were divided into control (vehicle, 10% DMSO), TBI, TBI + BAY (3 mg/kg, i.p), and BAY alone groups.	GFAP, Iba1, Mincle, Syk, NF-κB, TNF-α, IL-1β/ IL-6, Bax, Bim, ASC, and Caspase-1	BAY61-3606 treatment decreased levels of inflammatory markers and suppressed microglial activation. Additionally, the treatment attenuated TBI-induced neurovascular unit damage and improved neurological function impairments.	[70]
Weight-drop model	Levosimendan	In vivo	Wistar albino rats were divided into 4 groups: control without trauma, TBI without treatment, and levosimendan (i.p. injection at 12 μg/kg) administered 1 h or 2 h after the trauma. The weight-drop model was used as a TBI model.	Necrosis inhibition	Caspase-dependent apoptosis and necrosis were the main cell death processes after the TBIs. Levosimendan treatment exerted neuroprotection by reducing the number of necrotic cells in the brain tissue of the rats post-TBI.	[71]
Weight-drop model	TAT-KIR	In vivo	Male Kunming mice were divided into sham, TBI, and TBI+TAT-KIR groups. TAT-KIR was administered into the brain post-TBI surgery using defined stereotaxic coordinates.	GFAP, SOCS3, JAK2, and STAT3	GFAP levels were reduced after the treatment. The TAT-KIR treatment post-TBI in mice reduced harmful reactive astrocytes, inhibited the JAK2-STAT3 pathway, decreased neuron loss, and improved neural function, suggesting its potential as a neuroprotective therapy for TBIs.	[72]
LPS; FPI model	Abrocitinib	In vitro and in vivo	BV2 were first exposed to LPS; then, abrocitinib (100 nM, 500 nM, or 1 µM) was added to the culture medium. Fluid percussion injuries (FPIs) were performed on C57BL/6 mice. The mice were divided into sham, TBI+vehicle (saline, i.g.), and TBI+ abrocitinib (10 mg/kg, i.g.) groups.	Iba1, JAK1, STAT1, NF-κB, NLRP3, ASC/cleaved Caspase-1, GSDMD, IL-18, IL-1β, IL-6, and TNF-α	Abrocitinib promoted neuroprotection by reducing apoptosis and neuroinflammation. Abrocitinib reduced inflammation by inhibiting cell infiltration, microglia activation, and promoting M2 polarization, while decreasing pro-inflammatory cytokines. These effects were associated with decreased pyroptosis in brain tissues after the TBI by reducing the levels of NF-κB and its downstream factors.	[73]
Weight-drop model	SKEO and carvacrol	In vivo	Male Wistar rats were divided into the sham, TBI, TBI + vehicle, TBI+ carvacrol (100 or 200 mg/kg, i.p.), and TBI + SKEO200 (SKEO 200 mg/kg, i.p.) groups. All treatments and vehicles were administered 30 min after the TBI, and the rats were sacrificed 24 h after the TBI.	NF-κB, TNF-α, IL-1β, IL-6; cleaved Caspase 3, Bax, and Bcl-2	SKEO and carvacrol treatments reduced edema after TBI and modulated apoptotic-related proteins. Additionally, the levels of pro-inflammatory cytokines and pro-apoptotic markers were reduced after the treatments in TBI groups, resulting in the inhibition of neuronal damage.	[74]
	Papaverine	In vivo	Swiss Albino male mice were divided into sham, TBI+saline, TBI+papaverine, and papaverine alone groups. Papaverine was administered 30 min after the TBI with an i.p. injection of 40 mg/kg.	S100B, RAGE, and NF-κB	Serum S100B increased in the trauma group compared to the sham group, but the papaverine-treated TBI group showed a non-significant decrease. However, papaverine demonstrated properties that prevent cell death and protect nerve cells, likely through the RAGE-NF-κB pathway and potentially by reducing microglia activation in TBIs.	[75]
Weight-drop model	Saffron	In vivo	Male albino BABL/c mice were subjected to a rmTBI model. Mice were divided into sham, saffron + sham, TBI, and saffron + TBI groups. Saffron (50 mg/kg) was administered i.p. 30 min before injury.	GFAP, Iba1, NeuN, SIRT1, NF-κB, NLRP3, ASC, cleaved Caspase-1, GSDMD, IL-18, IL-1β, and Nrf2	Saffron extract upregulated SIRT1, Nrf2, and HO-1 and reduced NLRP3 inflammasome and cytokines. In addition, the treatment reduced GFAP, NeuN, and Iba1 in the injured cortex following rmTBI, indicating its potential effect on astrocytes, neurons, and microglia.	[76]
LPS model; CCI model	ACT001	In vitro and in vivo	The in vitro TBI model used co-cultures of BV2 cells with HT22 or bEnd.3 cells, treated with ACT001 (1–10 μM) and LPS (100 or 500 ng/mL). Adult male C57BL/6 mice subjected to a TBI received ACT001 through oral gavage (100 mg/kg) for 7 days. Rat primary cells were stimulated with LPS (1–500 μM), followed by ACT001 treatment.	Iba1, NeuN, AKT, NF-κB, NLRP3, Arg1, CD206, TGF-β and IL-10	ACT001 improved cognitive disfunction post-TBI, potentially by modulating microglial activation. It also attenuated neuroinflammation, decreased neuronal apoptosis by activated microglia, and mitigated the angiogenesis inhibition and tight junction in vitro. These effects were associated with reduced activation of the NF-κB and NLRP3 pathways via decreasing AKT phosphorylation.	[77]
CCI model	Genetic deletion of GSDMD	In vivo	GSDMD-KO and WT C57BL/6 mice were subjected to a TBI, with the sham groups serving as procedural controls. The CRISPR/Cas9 method was utilized to create the KO mice.	NLRP1, NLRP3, NLRC4, Caspase-1, GSDMD, IL-1β, TNF-α, IL-10, and TGF-β1	After the TBI, the NLRP3 inflammasome activated GSDMD, leading to pyroptosis. Moreover, GSDMS may be involved in microglial polarization. Inhibiting GSDMD showed neuroprotective effects, particularly in the early stages of the TBI, and resulted in reduced neuropathological changes and neurological impairments.	[78]
CCI model	H_2_S@SF	In vivo	Adult male ICR mice were treated with 20 μL SF or H_2_S@SF hydrogel 1 h after trauma at the injury site. Mice were divided into sham, TBI, SF, NaHS, H_2_S@SF, SF + TBI, NaHS + TBI, and H_2_S@SF + TBI groups.	GFAP, Iba1, NLRP3, Caspase-1, IL-1β, TNF-α, GDSMD, ASC, RIP-1, endogenous H_2_S pathway, and GSH	H_2_S^@^SF hydrogel inhibited neuronal pyroptosis and exerted neuroprotective effects against neuron loss and chronic neuroinflammation, promoting neurological recovery. The effects on neuroinflammation may be linked to the reduction in GFAP and Iba1 expression levels.	[79]
CCI model	Urolithin A	In vivo	Male C57BL/6 mice were divided into sham, TBI + vehicle, and TBI + urolithin A (2.5 mg/kg, i.p.) groups.	PI3K, AKT, mTOR, AKT, IKK, and NF-κB	Urolithin A was neuroprotective against the TBI by reducing the disruption of the BBB, cerebral edema, and neuronal apoptosis, and by improving autophagy and reducing neurological damage.	[80]
CCI model	COG1410	In vivo	Male C57BL/6 WT and TREM2 KO mice were divided into sham, TBI + vehicle, and TBI + COG1410 treatment via i.v. injection groups.	Iba1, TREM2, AKT, CREB, BDNF, TNF-α, and IL-1β	TREM2 activation by COG1410 reduced neural damage via the AKT/CREB/BDNF pathway in microglia, improving TBI outcomes.	[81]
LPS; CCI model	VU0360172	In vitro and in vivo	Primary rat microglia and BV2 cells were first treated with VU0360172 (20 or 50 µM), followed by LPS (100 ng/mL) for 24 h. siRNAs against mGluR5 and plasmids expressing CREB were used in BV2. C57BL/6J and CX3CR1^gfp/+^ mice were divided into sham, TBI + VU0360172, and TBI + vehicle groups.	PI3K, AKT, GSK-3β, CREB, iNOS, NO, TNF-α, IL-10, and Arg-1	VU0360172, a mGluR5-positive allosteric modulator, promoted neuroprotection post-TBI in mice, reducing pro-inflammatory micro-glial responses and enhancing anti-inflammatory phenotypes. These effects did not depend on NFκB activation in microglia.	[82]
LPS; stab wound cortical injury	Quinpirole	In vitro and in vivo	HT22 cells were co-treated with LPS-stimulated microglial medium (BV2 cells) for 24 h. Male C57BL/6 mice were divided into control (saline), SWI, and SWI + quinpirole groups. Quinpirole was administered for 7 days at 1 mg/kg/day via i.p.	GFAP, Iba1, D2R, AKT, GSK-3β, and IL-1β	Quinpirol, acting as a D2R agonist, exerted neuroprotective effects and led to reduced neuroinflammation, BBB disruption, and neurodegeneration. These activities were associated with decreased GFAP and Iba1 levels after treatment, leading to the suppression of astrocyte and microglial activation.	[83]
**Antioxidant strategies in TBI therapy**						
CCI model	Cofilin inhibitor	In vitro and in vivo	SH-SY5Y were co-treated with 200 μM, with or without 10 μM cofilin inhibitor, for 24 h. Male C57BL/6 mice were divided into sham, TBI + vehicle (4.9% DMSO, 4.9% Tween-20, and 88.9% of a solubilizing agent), and TBI + cofilin inhibitor (25 mg/kg) groups. Cofilin inhibitor was given via i.v. injection 4 h after TBI, followed by i.p. injections every 12 h for 3 days.	Cofilin, AKT, Bax, Caspase-3, Nrf2, SOD2, HMGB1, TNF-α, and iNOS	In TBI, cofilin inhibitors reduced microglial activation, and activated Nrf2 in neurons, reducing apoptosis and oxidative stress.	[84]
H_2_O_2_ and LPS exposure; CCI model	Oridonin	In vitro and in vivo	BV2, RAW264.7 and N2a cells were used for the in vitro experiments. Female mice were divided into sham, TBI, and TBI + Oridonin (20 mg/kg) groups. Oridonin or vehicle (DMSO and saline) was administered via i.p. injection 30 min post-TBI and every day until the last sacrifice (7 or 14 day).	GFAP, Iba1, AKT, GSK-3β, NLRP3, IL-1β, and Nrf2/HO-1	Oridin improved function and reduced TBI-induced neuropathological effects by activating the Nrf2 pathway, enhancing mitochondrial function and antioxidants, and decreasing oxidative stress-induced neuroinflammation. Moreover, Oridonin reduced glial activation, as shown by reduced GFAP and Iba1 levels.	[85]
CCI model	Astaxanthin	In vivo	Male C57BL/6 mice were divided into sham, TBI + vehicle, and TBI + Astaxanthin groups.	Nrf2, HO-1, and cleaved Caspase-3	Astaxanthin reduced TBI-induced apoptosis and improved neuromotor function through Nrf2/HO-1 activation.	[86]
CCI model	Calcitriol	In vitro and in vivo	Primary cortical neurons from mice were pre-treated for 6 h with chloroquine (25 μM) and then treated with calcitriol (10 nM, 100 nM, and 500 nM) for 24 h. Male CD1 Elite mice and Nr2 KO were divided into sham, TBI, and TBI + calcitriol (0.5, 1, or 3 µg/kg/day orally) groups.	Keap1, Nrf2, and LC3	Calcitriol’s neuroprotective effects in the context of TBI were mediated through the regulation of the autophagy and Nrf2 signaling pathways, which play critical roles in maintaining redox balance and protecting against oxidative damage.	[87]
H_2_O_2_ exposure; Weight-drop model	Astaxanthin	In vitro and in vivo	Primary cortical neurons from mice were grouped as follows: control, H_2_O_2_, H_2_O_2_ + astaxanthin (25, 50, and 100 μM), and H_2_O_2_ + 100 μM of astaxanthin + selisistat. Male C57BL/6 WT and Nrf2 KO mice were divided into sham + vehicle, sham + astaxanthin (25, 75 or 150 mg/kg), and TBI + astaxanthin + selisistat. Vehicle was olive oil or 1% DMSO dissolved in saline.	Bcl2, Bax and cleaved Caspase-3, SIRT1, Nrf2, Prx2, ASK1, and p-38	Astaxanthin offers significant neuroprotection in TBI by reducing oxidative stress and neuronal apoptosis.	[88]
Weight-drop model	Hydrogen-rich saline	In vivo	Male C57BL/6J mice were induced with a TBI and subjected to i.p. injections of saline (control) or hydrogen rich (5 mL/kg) every day for 72 h.	Nrf2, GSH, MDA, SOD, HO-1, RIP1, RIP3, TNF-α, IL-1β, IL-6, and NF-κB	Hydrogen-rich saline treatment improved neurological scores, enhanced neuronal survival, reduced brain ROS and inflammation markers, and decreased necroptosis after the TBI.	[89]
FPI model	Sevoflurane	In vivo	Male Sprague Dawley rats were categorized into four groups: sham, TBI, TBI + sevoflurane, and TBI + sevoflurane + zinc protoporphyrin. They received 3% sevoflurane in pure oxygen with a delivery flow rate of 4 L/min.	Nrf2, HO-1, and SOD	Administering sevoflurane right after the TBI may lead to synaptic restructuring and cognitive enhancement.	[90]
FPI model	Atorvastatin	In vivo	Male ICR mice were divided into sham, TBI, TBI + vehicle (saline), and TBI + atorvastatin (10 mg/kg/day) groups. Two experimental procedures were conducted by using WT and Nrf2 KO mice.	Nrf2, HO-1, caspase-3, GRP78, p-PERK, p-PERK, p-IRE1α, cleaved-ATF6, and CHOP	Atorvastatin mitigated ER stress-mediated apoptosis following the TBI by activating the Nrf2/HO-1 signaling pathway. This mechanism underlines its neuroprotective effects in the context of TBI treatment.	[91]
CCI model	Tri-combo of apocynin, tBHQ, and salubrinal	In vivo	C57BL/6 mice were divided into sham, vehicle (1% DMSO), and TBI + tri-combo groups. A combination of apocynin (5 or 10 mg/kg), tBHQ (12.5 or 25 mg/kg), and salubrinal (1.5 or 3 mg/kg; Millipore Sigma) was injected i.p. at 5 min or 3, 24, and 48 h after the TBI.	ER stress, oxidative stress, and inflammation	The post-TBI tri-combo treatment improved motor and cognitive functions and reduced lesion volume. It was effective in both sexes and even showed promise for aged subjects, indicating potential efficacy regardless of age or gender.	[92]
Weight-drop model	Naringenin	In vivo	Experiments were performed in ICR male mice using sham, TBI, TBI + vehicle, and TBI + naringenin. Naringenin was intraperitoneally injected 30 min after TBI, and then daily. Mice were sacrificed three days following the TBI.	GRP78, p-EIF2α, ATF4, CHOP, Bcl2/BAX ratio, cleaved Caspase-3, GPx, and MDA	Elevated protein levels linked to ER-stress were found in the perilesional cortex post-TBI. Naringenin provided neuroprotection by inhibiting ER stress-associated apoptosis and exerting antioxidant activity, leading to improved neurological function and reduced cerebral damage caused by the TBI.	[93]
**Glutamate modulators to enhancing neuronal survival**						
Glutamate and H_2_O_2_ exposure; CCI model	PHLPP inhibitor NSC74429	In vitro and in vivo	Primary rat neurons were exposed to glutamate (10 µM) or H_2_O_2_ (40 µM), and then treated with 25 µM or 50 µM inhibitors. Male Sprague Dawley rats and C57BL/6 mice received NSC74429 at 1 or 3 mg/kg doses. Rats were given 1.25 mL/kg i.v. in 24% DMSO/dextrose 5% in water. Mice received a 250 µL bolus, i.v. or i.p., in 5% DMSO/dextrose 5% in water.	Apoptosis, necrosis, and excitotoxicity	The experiments encompassed various models of brain damage, including apoptosis, excitotoxicity, and oxidative stress, and demonstrated that NSC74429 could protect brain cells from a range of cell death mechanisms.	[94]
Weight-drop model	H_2_S	In vivo	Sprague Dawley rats were divided into sham, TBI, TBI + vehicle, and TBI + NaHS (1 mmol/kg) groups. NaHS or vehicle was administered i.p. 30 min before TBI.	Caspase-3, Bax, Bcl-2, GLS2, MDA, SOD, GPx, HO-1, and p53	H_2_S reduced TBI-induced motor and spatial memory deficits, brain edema, and apoptosis. Additionally, H_2_S suppressed TBI-induced glutamate and oxidative stress.	[95]
Scratch injury; CCI model	Edonerpic maleate	In vitro and in vivo	Cortical neurons from pregnant rats were treated with edonerpic maleate at concentrations of 0.1, 1, and 10 μM post-injury and glutamate exposure (100 μM). Male Sprague Dawley rats were orally pretreated with edonerpic maleate at doses of 5, 20, or 30 mg/kg once daily for 3 weeks prior to CCI. Functional assessments were performed 24 h after the TBI.	CRMP2, NR2B, Arc, and GluR1	Edonerpic maleate reduced neurotoxicity, regulated glutamate receptors, preserved intracellular calcium balance, attenuated brain damage, and preserved long-term neurological function after the TBI.	[97]
**Antiferroptotic strategies enhance neurorepair in TBI**						
CCI model	Ruxolitinib	In vivo	Male C57BL/6 mice were divided into groups for three types of experiments: sham, vehicle + TBI, mice treated i.p. with a specific ferroptosis inhibitor (2 mg/kg), and mice treated i.p. with ruxolitinib at a dose of 0.44 mg/kg.	GPX4, COX-2, TfR1, and FTL	Ruxolitinib, a JAK inhibitor, exerted neuroprotective effects by inhibiting ferroptosis induced by the TBI.	[98]
CCI model	Polydatin	In vitro and in vivo	Neuro2A cells were treated with 60 μM polydatin and 10 μM hemin for 12 h. Male C57BL/6 mice were divided into sham, TBI, and TBI+polydatin groups. Polydatin was administered at a dose of 50 mg/kg (DMSO 0.01% in 5% Tween-80, 10% N–N-dimethyl acetamide, 35% PEG400, and 50% saline) 30 min after the TBI.	GPX4	GPX4 and GSH protein levels were decreased 24 h after TBI induction. Treatment with polydatin administered post-injury reduced the extent of cortical damage, improving neurological functions.	[99]
CCI model	Netrin-1	In vitro and in vivo	Mechanical stretch model was performed in vitro in SHSY5Y cells. The CCI model was performed on C57BL/6 mice. Mice were divided into sham, TBI, TBI+shNetri-1, and TBI+ groups.	Nrf2 and GPX4	Netrin-1 inhibited ferroptosis and improved neurological functions by promoting Nrf2 nuclear translocation and, consequently, the transcription of GPX4.	[100]
CCI model	Deferoxamine and dimethyl fumarate	In vivo	Male C57BL/6 mice WT and Nrf2 KO were treated with vehicle, deferoxamine (50 mg/kg/day i.p.), or dimethyl fumarate (50 mg/kg/day i.g.). Each group of mice were divided into sham and TBI treated.	Nrf2, FTH, FTL, xCT, GPX4, GSH, and FSP1	The loss of Nrf2 led to decreased FTH and FTL levels, with a consequent increase in free iron levels. Treatment with deferoxamine ameliorated ferroptosis after TBI, while dimethyl fumarate also improved neurological impairments post-TBI.	[101]
Scratch injury; CCI model	Melatonin	In vitro and in vivo	HT22 cells were transfected with FTH siRNA or untreated, and then subjected to a mechanical scratch assay at 72 h. Male C57BL/6 WT, FTH floxed, and FTH KO mice were divided into sham and TBI groups. Treatments included melatonin (10 mg/kg) or vehicle (0.5% DMSO), luzindole, and liproxstatin-1.	MT2 and FTH	Melatonin alleviated ferroptosis, reducing iron accumulation and neuronal damage in the ipsilateral cortex after the TBI, and improving cognitive function in vivo. The MT2 receptor and FTH were essential for melatonin’s neuroprotective effect both in vitro and in vivo. The lack of FTH resulted in cortical iron buildup, increasing susceptibility to TBI-induced ferroptosis.	[102]
Weight-drop model	Melatonin	In vivo	Mice were subjected to a head injury three times for inducing repeated mTBI using a 40 g weight dropped from 15 cm onto a specific area of the skull. Additionally, melatonin was administered to the mice as a treatment (10 mg/kg/day, i.p.) once daily.	Astrocyte activation	Melatonin treatment, when applied early after the TBI, enhanced cognitive function, improved neuronal activity, and reduced astrocyte reactivation.	[103]
CCI model	Melatonin	In vivo	Male Wistar rats were divided into sham, TBI, and TBI + melatonin groups. Melatonin was administered (10 mg/kg) via i.p. injection at different times (0.5 h, 1 h, 2 h, 3 h, and 4 h) after the TBI.	Caspase-3, Bcl-2, cytochrome c, PGC1-α, OXPHOS, Drp1, and ATP synthase	Melatonin treatment provided neuroprotection against the TBI by affecting mitochondria. It reduced mitochondrial fragmentation, thereby preventing nerve cell dysfunction and death.	[104]
CCI model	H_2_S	In vivo	Mice were categorized into sham, TBI+vehicle, TBI+NaHS, TBI+Wnt3a, and Tbi+Lip-1 groups. Experiments focused on ferroptosis, cognitive impairments, and the Wnt–H_2_S interaction in ferroptosis-induced TBI.	Wnt3a and GPX4	H_2_S exerted neuroprotection by partially restoring neurological deficits. Inhibition of ferroptosis may have been induced by H_2_S, perhaps via the Wnt/β-catenin pathway.	[105]
Weight-drop model	Anacardic acid	In vivo	ICR male mice were divided into sham, TBI, TBI + Fer-1, and TBI + anacardic acid groups. Fer-1 (2 mg/kg) and anacardic acid (100 mg/kg, i.p.) were administered after inducing a TBI, with anacardic acid given for 7 days post-injury.	TfR1, GPX4, IL-6, TNF-α, IL-1β, and CXCL1	Anacardic acid therapy could mitigate brain damage and BBB deterioration caused by TBI by inhibiting ferroptosis. It also reduced neuronal loss and neurodegeneration induced by TBIs through its anti-ferroptosis and anti-inflammatory properties.	[106]
**Multifaceted approaches to TBI treatment**						
	Pirfenidone	In vivo	Male Wistar rats were divided into control (only anesthesia and scalp incision), TBI (2 mL of 0.9% saline after trauma), and PIR groups. Mice of pirfinedone groups received 500 mg/kg/day of Pirfenex (Cipla, India) after trauma and on the day after via orogastric gavage.	NSE, S100B, and Caspase-3	TBI increased the levels of NSE, SB100B, and Caspase-3. The treatment reduced levels of biomarkers while not significantly affecting caspase-3 levels. Additionally, pirfinedone showed neuroprotective effects by decreasing neuroinflammatory markers and improving neurological deficits.	[107]
Weight-drop model	DMSO	In vivo	Male Wistar rats were divided into control (without trauma and treatment), TBI, and TBI + DMSO (67.5 mg/kg orally) groups for 21 days.	S100B, SOD, GPx, and CAT genes	S100B levels were reduced in the DMSO-treated group compared to the trauma group. DMSO treatment improved cognitive function in TBI rats by enhancing antioxidant defense, suppressing lipid peroxidation, and reducing oxidative stress and neuropathology.	[108]
Compressed gas model	Curcumin	In vivo	Mice were divided into sham, TBI, TBI + vehicle, and TBI + curcumin groups. Curcumin at 50 mg/kg was administered i.p. 15 min after the TBI.	BBB integrity, oxidative stress, inflammation, and apoptosis	The neuroprotective effects of curcumin in TBI were potentially due to its actions on BBB integrity, reduction of cerebral edema, mitigation of oxidative stress and inflammation, inhibition of neuronal apoptosis, and improvement in neurological functions.	[109]
CCI model	Hidrox^®^	In vivo	Two-month-old Sprague Dawley rats were divided into three groups: vehicle (saline), Hidrox^®^ (10 mg/kg), and sham. Hidrox^®^ was administered 1 h after the TBI and administered daily via gavage for a period of 4 weeks.	Tau, Nrf2, HO-1, SOD, GSH, NF-κB, IL-1β, TNF-α, and IL-6	Treatment with Hidrox^®^, an antioxidant and anti-inflammatory agent, has the potential to effectively reduce inflammation and may counteract neurodegenerative progression triggered by both primary and secondary injuries.	[110]
LPS/IFNγ-induced neuroinflammation Lateral FPI model	Levetiracetam; trichostatin A	In vitro and in vivo	The study tested Trichostatin A, validated with primary JAXC57BL/6J mouse neurons and BV-2 co-cultures, on Sprague Dawley rats with a TBI. Two cohorts were used: one for levetiracetam and trichostatin (1 mg/kg) monotherapies and another for higher dose levetiracetam (150 mg/kg) and combined therapy. Drugs were administered via injections or minipumps, tailored to each cohort’s objectives.	NFH, cell death, nitrite levels, and humoral immune response	NFH plasma levels were linked to the cortical lesion area. Despite reducing seizures, levetiracetam did not significantly decrease cortical lesion areas after the TBI. Trichostatin A was promising in vitro, but not in vivo, either alone or in combination.	[113]
CCI model	Immunocal^®^	In vivo	Male CD1 Elite mice were divided into sham, rmTBI, and Immunocal^®^ + rmTBI groups. Immunocal^®^ was administered prior to, during, and post-rmTBI. Five rmTBIs were administered at 48-h intervals across 10 days.	GFAP, Iba1, and NF-L	rmTBI caused extended gliosis and inflammation, neuronal damage, and redox imbalance. Immunocal^®^ supplementation significantly prevented astrogliosis in the rmTBI model and microgliosis in the rmmTBI model.	[114]

AIF1: allograft inflammatory factor 1; AKT: protein kinase B; ALC: acetyl-l-carnitine; ASC: apoptosis-associated speck-like protein containing a caspase-recruitment domain; ATF4: activating transcription factor 4; BAY61-3606: Syk inhibitor; BAX: bcl-2-like protein 4; Bcl-2: B-cell lymphoma 2; BDNF: brain-derived neurotrophic factor; Bim: BCL2-Like 11; CCE: cerebral cortex extract; CCI: controlled cortical impact; CCL11: C-C motif chemokine 11; CHI: controlled head injury; CHOP: C/EBP homologous protein; COG1410: apoE mimic peptide; COX-2: cyclooxygenase 2; CREB: cAMP response element-binding protein; CXCL1: C-X-C motif chemokine ligand 1; DMSO: dimethyl sulfoxide; ER: endoplasmic reticulum; Fer-1: ferrostatin-1; FPI: fluid percussion injury; FTH: heavy-chain ferritin; FTL: light-chain ferritin; GDNF: glial cell line-derived neurotrophic factor; GFAP: glial fibrillary acidic protein; GLS2: Glutaminase 2; GluR1: α-amino-3-hydroxy-5-methyl-4-isoxazolepropionic acid receptor 1; GPX4: glutathione peroxidase 4; GRIA1: Glutamate ionotropic receptor AMPA type subunit 1; GRP78: glucose-regulated protein-78; GSDMD: gasdermin D; GSK-3β: glycogen synthase kinase-3 beta; H_2_O_2_: hydrogen peroxide; H_2_S: hydrogen sulfide; H_2_S@SF: surface-fill H_2_S-releasing silk fibroin hydrogel; HMGB1: High Mobility Group Box 1; HO-1: hemeoxygenase-1; Iba1: ionized calcium-binding adaptor molecule 1; IKK: IL-1β: interleukin-1 beta; i.g.: intragastric; IL-4: interleukin-4; IL-6: interleukin-6; IL-10: interleukin-10; IL-18: interleukin-18; iNOS: Inducible Nitric Oxide Synthase; i.p.: intraperitoneal; i.v.: intravenous; JAK: Janus Kinase; JM-20: 3-ethoxycarbonyl-2-methyl-4-(2-nitrophenyl)-4,11-dihydro1H-pyrido [2,3-b][1,5]benzodiazepine; KO: knockout; LC3: Microtubule-associated protein 1A/1B-light chain 3; LPS: lipopolysaccharide; MAPT: microtubule-associated protein Tau; MDA: malondialdehyde, mGluR5: metabotropic glutamate receptor 5; Mincle: macrophage-inducible C-type lectin; mild TBI: mTBI; NaHS: sodium hydrosulfide; NeuN: neuronal nuclear protein; NFH: neurofilament heavy chain; NF-L: neurofilament light chain; NF-κB: nuclear factor-kappa B; NGF: nerve growth factor; NLRC4: NLR family CARD domain containing 4; NLRP3: NLR family pyrin domain containing 3; NR2B: N-methyl-d-aspartate receptor subunit 2B; Nrf2: nuclear factor erythroid 2–related factor 2; NSE: neuron-specific enolase; OGD/R: oxygen glucose deprivation/re-oxygenation; p.o.: per os; RAGE: receptor for advanced glycation endproducts; RIP1: receptor interacting serine/threonine kinase 1; rmTBI: repetitive mild traumatic brain injury; rmmTBI: repetitive mild-moderate TBI; ROS: reactive oxygen species; SIRT1: NAD-dependent protein deacetylase sirtuin-1; SKEO: *Satureja khuzistanica* essential oil; SOCS3: suppressor of cytokine signaling-3; STAT: signal transducer and activator of transcription; Syk: spleen tyrosine kinase; TBI: traumatic brain injury; TDP-43: TAR DNA-binding protein 43; TfR1: transferrin receptor 1 protein; TNF-α: tumor necrosis factor-alpha; TREM2: triggering receptor expressed on myeloid cells 2; TrkB: tyrosine kinase receptor B; Wnt3a: wingless-type MMTV integration site family, member 3A; and WT: wild type.

## References

[1] McKee A.C., Daneshvar D.H. (2015). The neuropathology of traumatic brain injury. Handb. Clin. Neurol..

[2] Wang K., Cui D., Gao L. (2016). Traumatic brain injury: A review of characteristics, molecular basis and management. Front. Biosci. Landmark.

[3] Morrow S.E., Pearson M. (2010). Management Strategies for Severe Closed Head Injuries in Children.

[4] Mioni G., Grondin S., Stablum F. (2014). Temporal dysfunction in traumatic brain injury patients: Primary or secondary impairment?. Front. Hum. Neurosci..

[5] Abdul-Muneer P., Chandra N., Haorah J. (2015). Interactions of oxidative stress and neurovascular inflammation in the pathogenesis of traumatic brain injury. Mol. Neurobiol..

[6] Quintard H., Patet C., Suys T., Marques-Vidal P., Oddo M. (2015). Normobaric hyperoxia is associated with increased cerebral excitotoxicity after severe traumatic brain injury. Neurocritical Care.

[7] Chen C., Zhong X., Smith D.K., Tai W., Yang J., Zou Y., Wang L.-L., Sun J., Qin S., Zhang C.-L. (2019). Astrocyte-specific deletion of sox2 promotes functional recovery after traumatic brain injury. Cereb. Cortex.

[8] Burda J.E., Bernstein A.M., Sofroniew M.V. (2016). Astrocyte roles in traumatic brain injury. Exp. Neurol..

[9] Maas A.I.R., Menon D.K., Manley G.T., Abrams M., Akerlund C., Andelic N., Aries M., Bashford T., Bell M.J., Bodien Y.G. (2022). Traumatic brain injury: Progress and challenges in prevention, clinical care, and research. Lancet Neurol..

[10] Galgano M., Toshkezi G., Qiu X., Russell T., Chin L., Zhao L.R. (2017). Traumatic brain injury: Current treatment strategies and future endeavors. Cell Transplant..

[11] Hacker D., Jones C.A., Yasin E., Preece S., Davies H., Hawkins A., Belli A., Paton E. (2023). Cognitive outcome after complicated mild traumatic brain injury: A literature review and meta-analysis. J. Neurotrauma.

[12] Haarbauer-Krupa J., Pugh M.J., Prager E.M., Harmon N., Wolfe J., Yaffe K. (2021). Epidemiology of chronic effects of traumatic brain injury. J. Neurotrauma.

[13] Dijkland S.A., Foks K.A., Polinder S., Dippel D.W.J., Maas A.I.R., Lingsma H.F., Steyerberg E.W. (2020). Prognosis in moderate and severe traumatic brain injury: A systematic review of contemporary models and validation studies. J. Neurotrauma.

[14] Renga V. (2021). Clinical evaluation and treatment of patients with postconcussion syndrome. Neurol. Res. Int..

[15] Jannace K., Pompeii L., Gimeno Ruiz de Porras D., Perkison W.B., Yamal J.M., Trone D.W., Rull R.P. (2023). Lifetime traumatic brain injury and risk of post-concussive symptoms in the millennium cohort study. J. Neurotrauma.

[16] McKee A.C., Stein T.D., Huber B.R., Crary J.F., Bieniek K., Dickson D., Alvarez V.E., Cherry J.D., Farrell K., Butler M. (2023). Chronic traumatic encephalopathy (cte): Criteria for neuropathological diagnosis and relationship to repetitive head impacts. Acta Neuropathol..

[17] Gardner R.C., Yaffe K. (2015). Epidemiology of mild traumatic brain injury and neurodegenerative disease. Mol. Cell. Neurosci..

[18] Hallock H., Mantwill M., Vajkoczy P., Wolfarth B., Reinsberger C., Lampit A., Finke C. (2023). Sport-related concussion: A cognitive perspective. Neurol. Clin. Pract..

[19] Ueda P., Pasternak B., Lim C.E., Neovius M., Kader M., Forssblad M., Ludvigsson J.F., Svanstrom H. (2023). Neurodegenerative disease among male elite football (soccer) players in sweden: A cohort study. Lancet Public Health.

[20] Ladak A.A., Enam S.A., Ibrahim M.T. (2019). A review of the molecular mechanisms of traumatic brain injury. World Neurosurg..

[21] Slavoaca D., Muresanu D., Birle C., Rosu O.V., Chirila I., Dobra I., Jemna N., Strilciuc S., Vos P. (2020). Biomarkers in traumatic brain injury: New concepts. Neurol. Sci. Off. J. Ital. Neurol. Soc. Ital. Soc. Clin. Neurophysiol..

[22] Hier D.B., Obafemi-Ajayi T., Thimgan M.S., Olbricht G.R., Azizi S., Allen B., Hadi B.A., Wunsch D.C. (2021). Blood biomarkers for mild traumatic brain injury: A selective review of unresolved issues. Biomark. Res..

[23] Nishimura K., Cordeiro J.G., Ahmed A.I., Yokobori S., Gajavelli S. (2022). Advances in traumatic brain injury biomarkers. Cureus.

[24] Gutierre M.U., Telles J.P.M., Welling L.C., Rabelo N.N., Teixeira M.J., Figueiredo E.G. (2021). Biomarkers for traumatic brain injury: A short review. Neurosurg. Rev..

[25] Li C., Chen S., Siedhoff H.R., Grant D., Liu P., Balderrama A., Jackson M., Zuckerman A., Greenlief C.M., Kobeissy F. (2023). Low-intensity open-field blast exposure effects on neurovascular unit ultrastructure in mice. Acta Neuropathol. Commun..

[26] Pham L., Wright D.K., O’Brien W.T., Bain J., Huang C., Sun M., Casillas-Espinosa P.M., Shah A.D., Schittenhelm R.B., Sobey C.G. (2021). Behavioral, axonal, and proteomic alterations following repeated mild traumatic brain injury: Novel insights using a clinically relevant rat model. Neurobiol. Dis..

[27] Bernick C., Shan G., Ritter A., Ashton N.J., Blennow K., Lantero-Rodriguez J., Snellman A., Zetterberg H. (2023). Blood biomarkers and neurodegeneration in individuals exposed to repetitive head impacts. Alzheimer’s Res. Ther..

[28] Kochanek P.M., Berger R.P., Bayir H., Wagner A.K., Jenkins L.W., Clark R.S. (2008). Biomarkers of primary and evolving damage in traumatic and ischemic brain injury: Diagnosis, prognosis, probing mechanisms, and therapeutic decision making. Curr. Opin. Crit. Care.

[29] Gan Z.S., Stein S.C., Swanson R., Guan S., Garcia L., Mehta D., Smith D.H. (2019). Blood biomarkers for traumatic brain injury: A quantitative assessment of diagnostic and prognostic accuracy. Front. Neurol..

[30] Korley F.K., Jain S., Sun X., Puccio A.M., Yue J.K., Gardner R.C., Wang K.K.W., Okonkwo D.O., Yuh E.L., Mukherjee P. (2022). Prognostic value of day-of-injury plasma gfap and uch-l1 concentrations for predicting functional recovery after traumatic brain injury in patients from the us track-tbi cohort: An observational cohort study. Lancet Neurol..

[31] Bazarian J.J., Welch R.D., Caudle K., Jeffrey C.A., Chen J.Y., Chandran R., McCaw T., Datwyler S.A., Zhang H., McQuiston B. (2021). Accuracy of a rapid glial fibrillary acidic protein/ubiquitin carboxyl-terminal hydrolase l1 test for the prediction of intracranial injuries on head computed tomography after mild traumatic brain injury. Acad. Emerg. Med. Off. J. Soc. Acad. Emerg. Med..

[32] Korley F.K., Datwyler S.A., Jain S., Sun X., Beligere G., Chandran R., Marino J.A., McQuiston B., Zhang H., Caudle K.L. (2021). Comparison of gfap and uch-l1 measurements from two prototype assays: The abbott i-stat and architect assays. Neurotrauma Rep..

[33] Biberthaler P., Musaelyan K., Krieg S., Meyer B., Stimmer H., Zapf J., von Matthey F., Chandran R., Marino J.A., Beligere G. (2021). Evaluation of acute glial fibrillary acidic protein and ubiquitin c-terminal hydrolase-l1 plasma levels in traumatic brain injury patients with and without intracranial lesions. Neurotrauma Rep..

[34] Lippa S.M., Gill J., Brickell T.A., Guedes V.A., French L.M., Lange R.T. (2022). Blood biomarkers predict future cognitive decline after military-related traumatic brain injury. Curr. Alzheimer Res..

[35] Whitehouse D.P., Monteiro M., Czeiter E., Vyvere T.V., Valerio F., Ye Z., Amrein K., Kamnitsas K., Xu H., Yang Z. (2022). Relationship of admission blood proteomic biomarkers levels to lesion type and lesion burden in traumatic brain injury: A center-tbi study. EBioMedicine.

[36] Kelmendi F.M., Morina A.A., Mekaj A.Y., Dragusha S., Ahmeti F., Alimehmeti R., Morina Q., Berisha M., Krasniqi B., Kerolli B. (2023). Ability of s100b to predict post-concussion syndrome in paediatric patients who present to the emergency department with mild traumatic brain injury. Br. J. Neurosurg..

[37] Blackwell L.S., Wali B., Xiang Y., Alawieh A., Sayeed I., Reisner A. (2023). Prognostic value of plasma biomarkers s100b and osteopontin in pediatric tbi: A prospective analysis evaluating acute and 6-month outcomes after mild to severe tbi. Biomedicines.

[38] Crichton A., Ignjatovic V., Babl F.E., Oakley E., Greenham M., Hearps S., Delzoppo C., Beauchamp M.H., Guerguerian A.M., Boutis K. (2021). Interleukin-8 predicts fatigue at 12 months post-injury in children with traumatic brain injury. J. Neurotrauma.

[39] Papa L., Walter A.E., Wilkes J.R., Clonts H.S., Johnson B., Slobounov S.M. (2022). Effect of player position on serum biomarkers during participation in a season of collegiate football. J. Neurotrauma.

[40] Oris C., Durif J., Rouzaire M., Pereira B., Bouvier D., Kahouadji S., Abbot M., Brailova M., Lehmann S., Hirtz C. (2023). Blood biomarkers for return to play after concussion in professional rugby players. J. Neurotrauma.

[41] Giza C.C., McCrea M., Huber D., Cameron K.L., Houston M.N., Jackson J.C., McGinty G., Pasquina P., Broglio S.P., Brooks A. (2021). Assessment of blood biomarker profile after acute concussion during combative training among us military cadets: A prospective study from the ncaa and us department of defense care consortium. JAMA Netw. Open.

[42] Lange R.T., Lippa S., Brickell T.A., Gill J., French L.M. (2023). Serum tau, neurofilament light chain, glial fibrillary acidic protein, and ubiquitin carboxyl-terminal hydrolase l1 are associated with the chronic deterioration of neurobehavioral symptoms after traumatic brain injury. J. Neurotrauma.

[43] Halbgebauer R., Halbgebauer S., Oeckl P., Steinacker P., Weihe E., Schafer M.K., Roselli F., Gebhard F., Huber-Lang M., Otto M. (2022). Neurochemical monitoring of traumatic brain injury by the combined analysis of plasma beta-synuclein, nfl, and gfap in polytraumatized patients. Int. J. Mol. Sci..

[44] Vorn R., Devoto C., Meier T.B., Lai C., Yun S., Broglio S.P., Mithani S., McAllister T.W., Giza C.C., Kim H.S. (2023). Are epb41 and alpha-synuclein diagnostic biomarkers of sport-related concussion? Findings from the ncaa and department of defense care consortium. J. Sport Health Sci..

[45] Guedes V.A., Lai C., Devoto C., Edwards K.A., Mithani S., Sass D., Vorn R., Qu B.X., Rusch H.L., Martin C.A. (2021). Extracellular vesicle proteins and micrornas are linked to chronic post-traumatic stress disorder symptoms in service members and veterans with mild traumatic brain injury. Front. Pharmacol..

[46] Guedes V.A., Lange R.T., Lippa S.M., Lai C., Greer K., Mithani S., Devoto C., Edwards K.A., Wagner C.L., Martin C.A. (2022). Extracellular vesicle neurofilament light is elevated within the first 12-months following traumatic brain injury in a u.S military population. Sci. Rep..

[47] Guedes V.A., Mithani S., Williams C., Sass D., Smith E.G., Vorn R., Wagner C., Lai C., Gill J., Hinson H.E. (2022). Extracellular vesicle levels of nervous system injury biomarkers in critically ill trauma patients with and without traumatic brain injury. Neurotrauma Rep..

[48] Beard K., Yang Z., Haber M., Flamholz M., Diaz-Arrastia R., Sandsmark D., Meaney D.F., Issadore D. (2021). Extracellular vesicles as distinct biomarker reservoirs for mild traumatic brain injury diagnosis. Brain Commun..

[49] Gonzalez-Ortiz F., Dulewicz M., Ashton N.J., Kac P.R., Zetterberg H., Andersson E., Yakoub Y., Hanrieder J., Turton M., Harrison P. (2023). Association of serum brain-derived tau with clinical outcome and longitudinal change in patients with severe traumatic brain injury. JAMA Netw. Open.

[50] Posti J.P., Hossain I., Takala R.S., Liedes H., Newcombe V., Outtrim J., Katila A.J., Frantzen J., Ala-Seppala H., Coles J.P. (2017). Glial fibrillary acidic protein and ubiquitin c-terminal hydrolase-l1 are not specific biomarkers for mild ct-negative traumatic brain injury. J. Neurotrauma.

[51] Chen H., Ding V.Y., Zhu G., Jiang B., Li Y., Boothroyd D., Rezaii P.G., Bet A.M., Paulino A.D., Weber A. (2022). Association between blood and computed tomographic imaging biomarkers in a cohort of mild traumatic brain injury patients. J. Neurotrauma.

[52] Bazarian J.J., Biberthaler P., Welch R.D., Lewis L.M., Barzo P., Bogner-Flatz V., Gunnar Brolinson P., Buki A., Chen J.Y., Christenson R.H. (2018). Serum gfap and uch-l1 for prediction of absence of intracranial injuries on head ct (alert-tbi): A multicentre observational study. Lancet Neurol..

[53] Loane D.J., Stoica B.A., Faden A.I. (2015). Neuroprotection for traumatic brain injury. Handb. Clin. Neurol..

[54] Butterfield M., Bodnar D., Williamson F., Parker L., Ryan G. (2023). Prevalence of secondary insults and outcomes of patients with traumatic brain injury intubated in the prehospital setting: A retrospective cohort study. Emerg. Med. J. EMJ.

[55] Hiskens M.I., Li K.M., Schneiders A.G., Fenning A.S. (2023). Repetitive mild traumatic brain injury-induced neurodegeneration and inflammation is attenuated by acetyl-l-carnitine in a preclinical model. Front. Pharmacol..

[56] Stocchetti N., Zanier E.R. (2016). Chronic impact of traumatic brain injury on outcome and quality of life: A narrative review. Crit. Care.

[57] Bozdemir E., Vigil F.A., Chun S.H., Espinoza L., Bugay V., Khoury S.M., Holstein D.M., Stoja A., Lozano D., Tunca C. (2021). Neuroprotective roles of the adenosine a(3) receptor agonist ast-004 in mouse model of traumatic brain injury. Neurotherapeutics.

[58] Barreto G.E., Gonzalez J., Ramirez D. (2023). Network pharmacology and topological analysis on tibolone metabolites and their molecular mechanisms in traumatic brain injury. Biomed. Pharmacother. = Biomed. Pharmacother..

[59] Wen W., Cheng J., Tang Y. (2024). Brain perivascular macrophages: Current understanding and future prospects. Brain A J. Neurol..

[60] Shanaki-Bavarsad M., Almolda B., Gonzalez B., Castellano B. (2022). Astrocyte-targeted overproduction of il-10 reduces neurodegeneration after tbi. Exp. Neurobiol..

[61] Furtado A.B.V., Goncalves D.F., Hartmann D.D., Courtes A.A., Cassol G., Nunez-Figueredo Y., Argolo D.S., do Nascimento R.P., Costa S.L., da Silva V.D.A. (2021). Jm-20 treatment after mild traumatic brain injury reduces glial cell pro-inflammatory signaling and behavioral and cognitive deficits by increasing neurotrophin expression. Mol. Neurobiol..

[62] Shi M., Mi L., Li F., Li Y., Zhou Y., Chen F., Liu L., Chai Y., Yang W., Zhang J. (2022). Fluvoxamine confers neuroprotection via inhibiting infiltration of peripheral leukocytes and m1 polarization of microglia/macrophages in a mouse model of traumatic brain injury. J. Neurotrauma.

[63] Ahmad R., Khan A., Rehman I.U., Lee H.J., Khan I., Kim M.O. (2022). Lupeol treatment attenuates activation of glial cells and oxidative-stress-mediated neuropathology in mouse model of traumatic brain injury. Int. J. Mol. Sci..

[64] Kumar A., Loane D.J. (2012). Neuroinflammation after traumatic brain injury: Opportunities for therapeutic intervention. Brain Behav. Immun..

[65] Wei G., Wang J., Wu Y., Zheng X., Zeng Y., Li Y., Chen X. (2021). Sirtuin 1 alleviates neuroinflammation-induced apoptosis after traumatic brain injury. J. Cell. Mol. Med..

[66] Shih R.H., Wang C.Y., Yang C.M. (2015). Nf-kappab signaling pathways in neurological inflammation: A mini review. Front. Mol. Neurosci..

[67] Gao Y., Zhang H., Wang J., Li F., Li X., Li T., Wang C., Li L., Peng R., Liu L. (2023). Annexin a5 ameliorates traumatic brain injury-induced neuroinflammation and neuronal ferroptosis by modulating the nf-kb/hmgb1 and nrf2/ho-1 pathways. Int. Immunopharmacol..

[68] Liu X.L., Sun D.D., Zheng M.T., Li X.T., Niu H.H., Zhang L., Zhou Z.W., Rong H.T., Wang Y., Wang J.W. (2023). Maraviroc promotes recovery from traumatic brain injury in mice by suppression of neuroinflammation and activation of neurotoxic reactive astrocytes. Neural Regen. Res..

[69] Ding W., Cai C., Zhu X., Wang J., Jiang Q. (2022). Parthenolide ameliorates neurological deficits and neuroinflammation in mice with traumatic brain injury by suppressing stat3/nf-kappab and inflammasome activation. Int. Immunopharmacol..

[70] He X., Huang Y., Liu Y., Zhang X., Yue P., Ma X., Miao Z., Long X., Yang Y., Wan X. (2022). Bay61-3606 attenuates neuroinflammation and neurofunctional damage by inhibiting microglial mincle/syk signaling response after traumatic brain injury. Int. J. Mol. Med..

[71] Aycan A., Oksuz E., Gonullu E., Kume T., Ergur B., Akyol M.E., Tas A., Kuyumcu F. (2022). A new approach in the treatment of traumatic brain injury: The effects of levosimendan on necrosis, apoptosis, and oxidative stress. World Neurosurg..

[72] Cai Z., Zhang Z., Zhang L., Tan R., Wang Y., Sun M., Hu X., Ge Q., An J., Lu H. (2023). The kinase inhibitory region of socs3 attenuates reactive astrogliosis and astroglial scar in mice after traumatic brain injury. J. Chem. Neuroanat..

[73] Li T., Li L., Peng R., Hao H., Zhang H., Gao Y., Wang C., Li F., Liu X., Chen F. (2022). Abrocitinib attenuates microglia-mediated neuroinflammation after traumatic brain injury via inhibiting the jak1/stat1/nf-kappab pathway. Cells.

[74] Abbasloo E., Amiresmaili S., Shirazpour S., Khaksari M., Kobeissy F., Thomas T.C. (2023). Satureja khuzistanica jamzad essential oil and pure carvacrol attenuate tbi-induced inflammation and apoptosis via nf-kappab and caspase-3 regulation in the male rat brain. Sci. Rep..

[75] Saglam E., Zirh S., Aktas C.C., Muftuoglu S.F., Bilginer B. (2021). Papaverine provides neuroprotection by suppressing neuroinflammation and apoptosis in the traumatic brain injury via rage- nf-<kappa>b pathway. J. Neuroimmunol..

[76] Shaheen M.J., Bekdash A.M., Itani H.A., Borjac J.M. (2021). Saffron extract attenuates neuroinflammation in rmtbi mouse model by suppressing nlrp3 inflammasome activation via sirt1. PLoS ONE.

[77] Cai L., Gong Q., Qi L., Xu T., Suo Q., Li X., Wang W., Jing Y., Yang D., Xu Z. (2022). Act001 attenuates microglia-mediated neuroinflammation after traumatic brain injury via inhibiting akt/nfkappab/nlrp3 pathway. Cell Commun. Signal. CCS.

[78] Du H., Li C.H., Gao R.B., Cen X.Q., Li P. (2022). Ablation of gsdmd attenuates neurological deficits and neuropathological alterations after traumatic brain injury. Front. Cell. Neurosci..

[79] Chen X., Huang X., Liu C., Li S., Yang Z., Zhang F., Chen X., Shan H., Tao L., Zhang M. (2022). Surface-fill h(2)s-releasing silk fibroin hydrogel for brain repair through the repression of neuronal pyroptosis. Acta Biomater..

[80] Gong Q.Y., Cai L., Jing Y., Wang W., Yang D.X., Chen S.W., Tian H.L. (2022). Urolithin a alleviates blood-brain barrier disruption and attenuates neuronal apoptosis following traumatic brain injury in mice. Neural Regen. Res..

[81] Yan J., Zhang Y., Wang L., Li Z., Tang S., Wang Y., Gu N., Sun X., Li L. (2022). Trem2 activation alleviates neural damage via akt/creb/bdnf signalling after traumatic brain injury in mice. J. Neuroinflammation.

[82] Bhat S.A., Henry R.J., Blanchard A.C., Stoica B.A., Loane D.J., Faden A.I. (2021). Enhanced akt/gsk-3beta/creb signaling mediates the anti-inflammatory actions of mglur5 positive allosteric modulators in microglia and following traumatic brain injury in male mice. J. Neurochem..

[83] Alam S.I., Jo M.G., Park T.J., Ullah R., Ahmad S., Rehman S.U., Kim M.O. (2021). Quinpirole-mediated regulation of dopamine d2 receptors inhibits glial cell-induced neuroinflammation in cortex and striatum after brain injury. Biomedicines.

[84] Bahader G.A., James A.W., Almarghalani D.A., Shah Z.A. (2023). Cofilin inhibitor protects against traumatic brain injury-induced oxidative stress and neuroinflammation. Biology.

[85] Zhao X.J., Zhu H.Y., Wang X.L., Lu X.W., Pan C.L., Xu L., Liu X., Xu N., Zhang Z.Y. (2022). Oridonin ameliorates traumatic brain injury-induced neurological damage by improving mitochondrial function and antioxidant capacity and suppressing neuroinflammation through the nrf2 pathway. J. Neurotrauma.

[86] Gao F., Wu X., Mao X., Niu F., Zhang B., Dong J., Liu B. (2021). Astaxanthin provides neuroprotection in an experimental model of traumatic brain injury via the nrf2/ho-1 pathway. Am. J. Transl. Res..

[87] Cui C., Wang C., Jin F., Yang M., Kong L., Han W., Jiang P. (2021). Calcitriol confers neuroprotective effects in traumatic brain injury by activating nrf2 signaling through an autophagy-mediated mechanism. Mol. Med..

[88] Zhang X.S., Lu Y., Li W., Tao T., Peng L., Wang W.H., Gao S., Liu C., Zhuang Z., Xia D.Y. (2021). Astaxanthin ameliorates oxidative stress and neuronal apoptosis via sirt1/nrf2/prx2/ask1/p38 after traumatic brain injury in mice. Br. J. Pharmacol..

[89] Hu Y., Feng X., Chen J., Wu Y., Shen L. (2022). Hydrogen-rich saline alleviates early brain injury through inhibition of necroptosis and neuroinflammation via the ros/ho-1 signaling pathway after traumatic brain injury. Exp. Ther. Med..

[90] Li C., Yu T.Y., Gong L.R., Mu R., Zhang Y., Yu J.B. (2021). Involvement of nrf-2/ho-1 pathway in sevoflurane-induced cognitive improvement in rats with traumatic brain injury. Behav. Brain Res..

[91] Feng Y., Lang J., Sun B., Yan Z., Zhao Z., Sun G. (2023). Atorvastatin prevents endoplasmic reticulum stress-mediated apoptosis via the nrf2/ho-1 signaling pathway in tbi mice. Neurol. Res..

[92] Davis C.K., Bathula S., Hsu M., Morris-Blanco K.C., Chokkalla A.K., Jeong S., Choi J., Subramanian S., Park J.S., Fabry Z. (2022). An antioxidant and anti-er stress combo therapy decreases inflammation, secondary brain damage and promotes neurological recovery following traumatic brain injury in mice. J. Neurosci. Off. J. Soc. Neurosci..

[93] Deng C., Yi R., Fei M., Li T., Han Y., Wang H. (2021). Naringenin attenuates endoplasmic reticulum stress, reduces apoptosis, and improves functional recovery in experimental traumatic brain injury. Brain Res..

[94] Jackson T.C., Dezfulian C., Vagni V.A., Stezoski J., Janesko-Feldman K., Kochanek P.M. (2022). Phlpp inhibitor nsc74429 is neuroprotective in rodent models of cardiac arrest and traumatic brain injury. Biomolecules.

[95] Sun J., Li X., Gu X., Du H., Zhang G., Wu J., Wang F. (2021). Neuroprotective effect of hydrogen sulfide against glutamate-induced oxidative stress is mediated via the p53/glutaminase 2 pathway after traumatic brain injury. Aging.

[96] Lim S.W., Su H.C., Nyam T.E., Chio C.C., Kuo J.R., Wang C.C. (2021). Ceftriaxone therapy attenuates brain trauma in rats by affecting glutamate transporters and neuroinflammation and not by its antibacterial effects. BMC Neurosci..

[97] Chen T., Yang L.K., Ai P., Zhu J., Hang C.H., Wang Y.H. (2022). Edonerpic maleate regulates glutamate receptors through crmp2- and arc-mediated mechanisms in response to brain trauma. Cell Death Discov..

[98] Chen X., Gao C., Yan Y., Cheng Z., Chen G., Rui T., Luo C., Gao Y., Wang T., Chen X. (2021). Ruxolitinib exerts neuroprotection via repressing ferroptosis in a mouse model of traumatic brain injury. Exp. Neurol..

[99] Huang L., He S., Cai Q., Li F., Wang S., Tao K., Xi Y., Qin H., Gao G., Feng D. (2021). Polydatin alleviates traumatic brain injury: Role of inhibiting ferroptosis. Biochem. Biophys. Res. Commun..

[100] Zhang Y., Lan J., Zhao D., Ruan C., Zhou J., Tan H., Bao Y. (2023). Netrin-1 upregulates gpx4 and prevents ferroptosis after traumatic brain injury via the unc5b/nrf2 signaling pathway. CNS Neurosci. Ther..

[101] Cheng H., Wang P., Wang N., Dong W., Chen Z., Wu M., Wang Z., Yu Z., Guan D., Wang L. (2023). Neuroprotection of nrf2 against ferroptosis after traumatic brain injury in mice. Antioxidants.

[102] Rui T., Wang H., Li Q., Cheng Y., Gao Y., Fang X., Ma X., Chen G., Gao C., Gu Z. (2021). Deletion of ferritin h in neurons counteracts the protective effect of melatonin against traumatic brain injury-induced ferroptosis. J. Pineal Res..

[103] Cao R., Li L., Zhang W., Lu J., Wang Y., Chen Q., Zhang W., Chen M., Sheng L., Cai K. (2021). Melatonin attenuates repeated mild traumatic brain injury-induced cognitive deficits by inhibiting astrocyte reactivation. Biochem. Biophys. Res. Commun..

[104] Salman M., Kaushik P., Tabassum H., Parvez S. (2021). Melatonin provides neuroprotection following traumatic brain injury-promoted mitochondrial perturbation in wistar rat. Cell. Mol. Neurobiol..

[105] Chen J., Chen Z., Yu D., Yan Y., Hao X., Zhang M., Zhu T. (2023). Neuroprotective effect of hydrogen sulfide subchronic treatment against tbi-induced ferroptosis and cognitive deficits mediated through wnt signaling pathway. Cell. Mol. Neurobiol..

[106] Liu Y., Zhao Z., Guo J., Ma Y., Li J., Ji H., Chen Z., Zheng J. (2023). Anacardic acid improves neurological deficits in traumatic brain injury by anti-ferroptosis and anti-inflammation. Exp. Neurol..

[107] Bozkurt I., Ozturk Y., Guney G., Arslan B., Gulbahar O., Guvenc Y., Senturk S., Yaman M.E. (2022). Effects of pirfenidone on experimental head injury in rats. Int. J. Clin. Exp. Pathol..

[108] Bulama I., Nasiru S., Bello A., Abbas A.Y., Nasiru J.I., Saidu Y., Chiroma M.S., Mohd Moklas M.A., Mat Taib C.N., Waziri A. (2022). Antioxidant-based neuroprotective effect of dimethylsulfoxide against induced traumatic brain injury in a rats model. Front. Pharmacol..

[109] Chen B., Shi Q.X., Nie C., Zhao Z.P., Wang T., Zhou Q., Gu J. (2023). Curcumin alleviates oxidative stress, neuroinflammation, and promotes behavioral recovery after traumatic brain injury. Curr. Neurovascular Res..

[110] Cordaro M., Trovato Salinaro A., Siracusa R., D’Amico R., Impellizzeri D., Scuto M., Ontario M.L., Crea R., Cuzzocrea S., Di Paola R. (2021). Hidrox((r)) roles in neuroprotection: Biochemical links between traumatic brain injury and alzheimer’s disease. Antioxidants.

[111] Atwood R., Walker P., Walper D., Elster E., Bradley M. (2023). Use of levetiracetam for post-traumatic seizure prophylaxis in combat-related traumatic brain injury. Mil. Med..

[112] Hedges A., Findlay M.C., Davis G.E., Wolfe B.M., Hawryluk G.W.J., Menacho S.T., Ansari S. (2023). Levetiracetam dosing for seizure prophylaxis in neurocritical care patients. Brain Inj..

[113] Kajevu N., Lipponen A., Andrade P., Banuelos I., Puhakka N., Hamalainen E., Natunen T., Hiltunen M., Pitkanen A. (2023). Treatment of status epilepticus after traumatic brain injury using an antiseizure drug combined with a tissue recovery enhancer revealed by systems biology. Int. J. Mol. Sci..

[114] Koza L.A., Pena C., Russell M., Smith A.C., Molnar J., Devine M., Serkova N.J., Linseman D.A. (2023). Immunocal(r) limits gliosis in mouse models of repetitive mild-moderate traumatic brain injury. Brain Res..

[115] Friedenstein A., Chailakhjan R., Lalykina K. (1970). The development of fibroblast colonies in monolayer cultures of guinea-pig bone marrow and spleen cells. Cell Prolif..

[116] Toupet K., Maumus M., Luz-Crawford P., Lombardo E., Lopez-Belmonte J., van Lent P., Garin M.I., van den Berg W., Dalemans W., Jorgensen C. (2015). Survival and biodistribution of xenogenic adipose mesenchymal stem cells is not affected by the degree of inflammation in arthritis. PLoS ONE.

[117] Li J., Zhu H., Liu Y., Li Q., Lu S., Feng M., Xu Y., Huang L., Ma C., An Y. (2010). Human mesenchymal stem cell transplantation protects against cerebral ischemic injury and upregulates interleukin-10 expression in macaca fascicularis. Brain Res..

[118] Xin H., Li Y., Shen L.H., Liu X., Wang X., Zhang J., Pourabdollah-Nejad D.S., Zhang C., Zhang L., Jiang H. (2010). Increasing tpa activity in astrocytes induced by multipotent mesenchymal stromal cells facilitate neurite outgrowth after stroke in the mouse. PLoS ONE.

[119] Zanier E.R., Pischiutta F., Riganti L., Marchesi F., Turola E., Fumagalli S., Perego C., Parotto E., Vinci P., Veglianese P. (2014). Bone marrow mesenchymal stromal cells drive protective m2 microglia polarization after brain trauma. Neurotherapeutics.

[120] Peruzzaro S., Andrews M., Al-Gharaibeh A., Pupiec O., Resk M., Story D., Maiti P., Rossignol J., Dunbar G. (2019). Transplantation of mesenchymal stem cells genetically engineered to overexpress interleukin-10 promotes alternative inflammatory response in rat model of traumatic brain injury. J. Neuroinflammation.

[121] Pischiutta F., D’Amico G., Dander E., Biondi A., Biagi E., Citerio G., De Simoni M.G., Zanier E.R. (2014). Immunosuppression does not affect human bone marrow mesenchymal stromal cell efficacy after transplantation in traumatized mice brain. Neuropharmacology.

[122] Pischiutta F., Brunelli L., Romele P., Silini A., Sammali E., Paracchini L., Marchini S., Talamini L., Bigini P., Boncoraglio G.B. (2016). Protection of brain injury by amniotic mesenchymal stromal cell-secreted metabolites. Crit. Care Med..

[123] Gutiérrez-Fernández M., Rodríguez-Frutos B., Ramos-Cejudo J., Teresa Vallejo-Cremades M., Fuentes B., Cerdán S., Díez-Tejedor E. (2013). Effects of intravenous administration of allogenic bone marrow-and adipose tissue-derived mesenchymal stem cells on functional recovery and brain repair markers in experimental ischemic stroke. Stem Cell Res. Ther..

[124] Xu Y., Hu Y., Xu S., Liu F., Gao Y. (2021). Exosomal micrornas as potential biomarkers and therapeutic agents for acute ischemic stroke: New expectations. Front. Neurol..

[125] Wang J., Wang J., Li X., Shu K. (2022). Cell-derived exosomes as therapeutic strategies and exosome-derived micrornas as biomarkers for traumatic brain injury. J. Clin. Med..

[126] Vaughn M.N., Winston C.N., Levin N., Rissman R.A., Risbrough V.B. (2021). Developing biomarkers of mild traumatic brain injury: Promise and progress of cns-derived exosomes. Front. Neurol..

[127] Fan Y., Chen Z., Zhang M. (2022). Role of exosomes in the pathogenesis, diagnosis, and treatment of central nervous system diseases. J. Transl. Med..

[128] Tang L., Xu Y., Wang L., Pan J. (2023). Adipose-derived stem cell exosomes ameliorate traumatic brain injury through the nlrp3 signaling pathway. Neuroreport.

[129] Zhang L., Lin Y., Bai W., Sun L., Tian M. (2023). Human umbilical cord mesenchymal stem cell-derived exosome suppresses programmed cell death in traumatic brain injury via pink1/parkin-mediated mitophagy. CNS Neurosci. Ther..

[130] Zhang L., Bai W., Peng Y., Lin Y., Tian M. (2024). Human umbilical cord mesenchymal stem cell-derived exosomes provide neuroprotection in traumatic brain injury through the lncrna tubb6/nrf2 pathway. Brain Res..

[131] Fricker M., Tolkovsky A.M., Borutaite V., Coleman M., Brown G.C. (2018). Neuronal cell death. Physiol. Rev..

[132] Zhang W., Hong J., Zhang H., Zheng W., Yang Y. (2021). Astrocyte-derived exosomes protect hippocampal neurons after traumatic brain injury by suppressing mitochondrial oxidative stress and apoptosis. Aging.

[133] DeWitt D.S., Hawkins B.E., Dixon C.E., Kochanek P.M., Armstead W., Bass C.R., Bramlett H.M., Buki A., Dietrich W.D., Ferguson A.R. (2018). Pre-clinical testing of therapies for traumatic brain injury. J. Neurotrauma.

[134] Zhao Q., Zhang J., Li H., Li H., Xie F. (2023). Models of traumatic brain injury-highlights and drawbacks. Front. Neurol..

[135] Wiegand T.L.T., Sollmann N., Bonke E.M., Umeasalugo K.E., Sobolewski K.R., Plesnila N., Shenton M.E., Lin A.P., Koerte I.K. (2022). Translational neuroimaging in mild traumatic brain injury. J. Neurosci. Res..

